# Lactylated Apolipoprotein C‐II Induces Immunotherapy Resistance by Promoting Extracellular Lipolysis

**DOI:** 10.1002/advs.202406333

**Published:** 2024-07-09

**Authors:** Jian Chen, Deping Zhao, Yupeng Wang, Ming Liu, Yuan Zhang, Tingting Feng, Chao Xiao, Huan Song, Rui Miao, Li Xu, Hongwei Chen, Xiaoying Qiu, Yi Xu, Jingxuan Xu, Zelin Cui, Wei Wang, Yanchun Quan, Yifeng Zhu, Chen Huang, Song Guo Zheng, Jian‐yuan Zhao, Ting Zhu, Lianhui Sun, Guangjian Fan

**Affiliations:** ^1^ Precision Research Center for Refractory Diseases, Institute for Clinical Research Shanghai General Hospital Shanghai Jiao Tong University School of Medicine Shanghai 200080 P. R. China; ^2^ Department of Thoracic Surgery, Shanghai Pulmonary Hospital Tongji University 507 Zhengmin Road Shanghai 200433 P. R. China; ^3^ Department of General Surgery, Shanghai Ninth People's Hospital Shanghai JiaoTong University School of Medicine Shanghai 200011 P. R. China; ^4^ Department of Gastrointestinal Surgery Shanghai General Hospital Shanghai Jiao Tong University School of Medicine Shanghai 201620 P. R. China; ^5^ Department of Clinical Pharmacy Shanghai General Hospital Shanghai Jiao Tong University School of Medicine Shanghai 201620 P. R. China; ^6^ Department of Gastrointestinal Surgery Shanghai East Hospital, School of Medicine Tongji University Shanghai 200040 P. R. China; ^7^ Department of Clinical Laboratory Medicine Shanghai Pulmonary Hospital Tongji University School of Medicine Shanghai 200433 P. R. China; ^8^ Institute of Transfusion Medicine and Immunology, Mannheim Institute of Innate Immunosciences (MI3), Medical Faculty Mannheim Heidelberg University 68167 Mannheim Germany; ^9^ Department of Laboratory Medicine Shanghai General Hospital Shanghai Jiao Tong University School of Medicine Shanghai 201620 P. R. China; ^10^ Department of Breast‐thyroid Surgery Shanghai General Hospital Shanghai Jiao Tong University School of Medicine Shanghai 201620 P. R. China; ^11^ Central Laboratory Linyi People's Hospital Shandong 273300 P. R. China; ^12^ Department of Internal Medicine II, Klinikum rechts der Isar Technical University of Munich Ismaninger Str. 22 81675 Munich Germany; ^13^ Department of Immunology, School of Cell and Gene Therapy, Songjiang Research Institute Shanghai Jiaotong University School of Medicine Affiliated Songjiang Hospital Shanghai 200080 P. R. China; ^14^ Institute for Developmental and Regenerative Cardiovascular Medicine, MOE‐Shanghai Key Laboratory of Children's Environmental Health Xinhua Hospital Shanghai Jiao Tong University School of Medicine Shanghai 200092 P. R. China

**Keywords:** APOC2, lipolysis, lysine‐lactylation, non‐small cell lung cancer

## Abstract

Mortality rates due to lung cancer are high worldwide. Although PD‐1 and PD‐L1 immune checkpoint inhibitors boost the survival of patients with non‐small‐cell lung cancer (NSCLC), resistance often arises. The Warburg Effect, which causes lactate build‐up and potential lysine‐lactylation (Kla), links immune dysfunction to tumor metabolism. The role of non‐histone Kla in tumor immune microenvironment and immunotherapy remains to be clarified. Here, global lactylome profiling and metabolomic analyses of samples from patients with NSCLC is conducted. By combining multi‐omics analysis with in vitro and in vivo validation, that intracellular lactate promotes extracellular lipolysis through lactyl‐APOC2 is revealed. Mechanistically, lactate enhances APOC2 lactylation at K70, stabilizing it and resulting in FFA release, regulatory T cell accumulation, immunotherapy resistance, and metastasis. Moreover, the anti‐APOC2^K70‐lac^ antibody that sensitized anti‐PD‐1 therapy in vivo is developed. This findings highlight the potential of anti lactyl‐APOC2‐K70 approach as a new combination therapy for sensitizing immunotherapeutic responses.

## Graphical abstract

Although PD‐1/PD‐L1 immune‐checkpoint inhibitors boost the survival of patients with NSCLC, resistance often arises. Combining global lactylome profiling, metabolomics and mechanistic‐experiments, it is found that intracellular lactate promotes extracellular lipolysis through lactyl‐APOC2‐K70, inducing Tregs accumulation and immunotherapy resistance. An anti‐APOC2^K70‐lac^ antibody that sensitized anti‐PD‐1 therapy in vivo is developed. This findings suggest that targeting lactylated APOC2‐K70 could improve the effectiveness of immunotherapy.

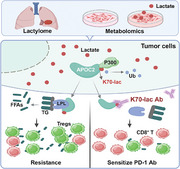

## 1 Introduction

Lung cancer is the leading cause of death worldwide. Immune checkpoint inhibitors targeting Programmed cell Death protein 1 (PD‐1) and Programmed Death‐Ligand 1 (PD‐L1) have improved the survival of patients with non‐small cell lung cancer (NSCLC). However, 20%−44% of patients with lung cancer receiving immunotherapy develop resistance. One of the main reasons for this is metabolic reprogramming during treatment.^[^ ^1^, ^2^, ^3^ ^]^ In the realm of immune function regulation, substantial focus has been directed toward the exploration of metabolites and metabolic pathways.^[^ ^4^, ^5^, ^6^ ^]^ Lactate and free fatty acids (FFAs) are typical small‐molecule metabolites present at high concentrations in the tumor microenvironment (TME). While previous reports have suggested that they can directly provide energy to various cells in tumor tissues, leading to tumor progression and resistance,^[^ ^7^, ^8^ ^]^ a deeper understanding of the potential and intricate orchestration of interactions between lactate, FFAs, and relevant metabolic factors could yield novel insights into tumor progression and open new avenues for treatment.

Lactate‐derived lactylation has emerged as a novel form of post‐translational modification (PTM),^[^ ^30^ ^]^ specifically affecting lysine residues (K) of histone proteins. Under conditions of elevated lactate levels, histone lysine is subject to lactylation, a process known to underlie various pathological processes, such as macrophage polarization^[^ ^30^, ^32^ ^]^ and tumorigenesis.^[^ ^33^ ^]^ Non‐histone lysine lactylation has been identified in hepatocellular cancer, where it promotes tumor proliferation and metastasis.^[^ ^9^ ^]^ This underscores the pivotal role of lysine lactylation as a critical nexus bridging lactate metabolism, tumor metabolism, and patient outcomes. Our previous study confirmed that non‐histone lactylation protein METTL16 augments the therapeutic efficacy by promoting cuproptosis.^[^ ^10^ ^]^ However, the implications of non‐histone lysine lactylation within the TME onimmunotherapy outcomes and the prognostic landscape for patients with cancer remain an area yet to be systematically investigated.

Overactive lipid metabolism is a typical feature of tumors and a key driver of tumor progression. In terms of tumor treatment resistance, especially to immunotherapy, the key role of glycolipid metabolism remains to be elucidated. The typical molecule in overactive lipid metabolism, FFAs are a source of energy for tumor cells, and participate in the formation of tumor cell membranes and the production of signaling molecules.^[^ ^7^, ^11^, ^12^ ^]^ Furthermore, FFAs are another relevant energy source for immune cells in the TME, including supporting regulatory T (Treg) cell accumulation in the low‐glucose TME of a subset of gastric cancer mutation, and generating an immunosuppressive TME that underlies resistance to immune checkpoint blockade.^[^ ^7^ ^]^ Consequently, strategies aimed at curbing FFA generation within the TME are attractive adjunct interventions. By modulating immune cell function through these means, the potency of immunotherapeutic approaches can be optimized for maximal efficacy.

In this study, numerous non‐histone Kla sites were identified in NSCLC, correlating with tumor metastasis and immunotherapy resistance. Combining with metabolomic analyses, we revealed that lactate induces lactyl‐APOC2‐K70, triggering the release of FFAs into the extracellular space and enhancing Treg cell accumulation. This interplay contributes to immunotherapy resistance and tumor metastasis. We validated these findings using antibody targeting lactyl‐APOC2‐K70 in combination with PD‐1 antibody in vivo, which showed a significant reduction in immunotherapy resistance. Overall, our findings emphasize the role of non‐histone lysine lactylation in tumor progression, offering potential biomarkers for predicting immunotherapy resistance and suggesting a promising therapeutic strategy for NSCLC.

## 2 Results

### 2.1 Characterization of the Lactylome in Non‐Small Cell Lung Cancer

**Figure 1:**
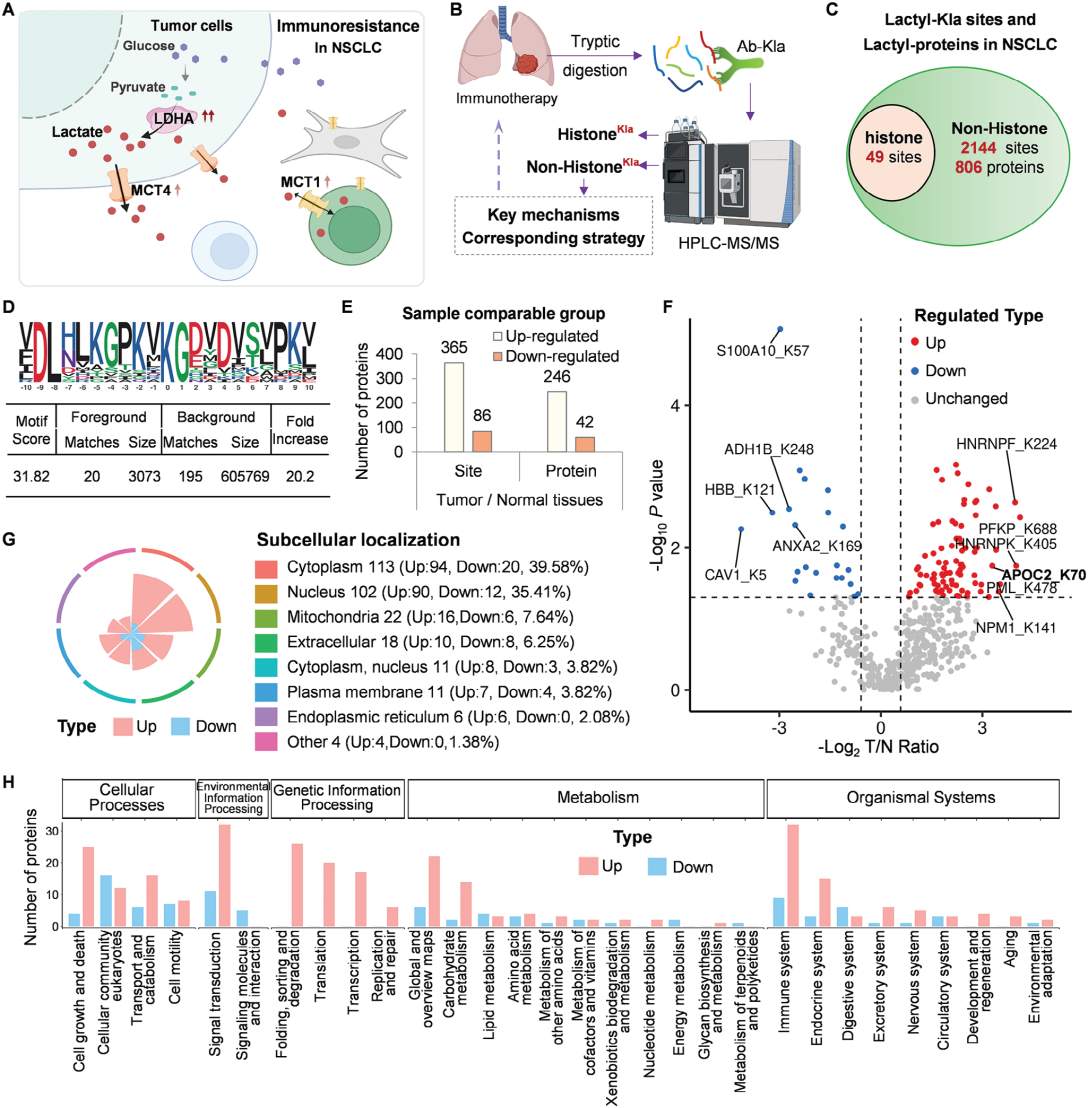
Characterization of the lactylome in Lung cancer. A) Simplified Diagram of Lactic Acid Metabolism and the correlation between MCT1, MCT4, or LDHA and patients with major pathologic response (MPR) in NSCLC. ↑ *p* < 0.05, ↑↑ *p* < 0.01, Student's t‐test. The staining of MCT1, MCT4, or LDHA in 40 samples of NSCLC was calculated on a scale of 1–12 points according to the staining of IHC in a double‐blinded manner (Figure S1B–E, Supporting Information). B) The whole flow chart of total Lysine lactylation via HPLC‐MS/MS in NSCLC. C) The numbers of total Kla sites and lactylated histone and non‐histone proteins. D) Sequence motif logo displaying a representative sequence for all Kla sites. E) Histogram indicating upregulated or downregulated lactyl‐proteins in tumor versus adjacent tissues. F) Volcano plot illustrating differential expression of Kla sites in tumors compared to adjacent tissues. The two vertical dashed lines and one horizontal dashed line denote the cutoff values for log_2_ of tumor/ adjacent ratio (1 and −1) and FDR (0.05), respectively. G) Cellular compartment distribution of differentially expressed lactyl‐proteins (numbers and percentages) in tumors compared with adjacent tissues. H) Histogram presenting the number of up‐ or down‐ regulated lactyl‐proteins in different pathways, classified according to the KEGG map, in tumors and adjacent tissues.

Lactate and its lactylation modifications are becoming increasingly important for cancer treatment. To explore the roles of lactate and lactylation in tumor treatment, we selected forty NSCLC clinical specimens from patients who underwent a combination of immunotherapy and chemotherapy (Figure S1A, Supporting Information). Lactate synthesis and transportation are mainly mediated by lactate dehydrogenase A (LDHA, synthesis),^[^ ^13^ ^]^ monocarboxylate transporter 4 (MCT4, exporter),^[^ ^14^ ^]^ and monocarboxylate transporter 1 (MCT1, transporter).^[^ ^15^ ^]^ We evaluated the protein levels of LDHA, MCT1, and MCT4 in these samples and found that they were associated with resistance to therapy (**Figure** **1**A; Figure S1B–E, Supporting Information). Intriguingly, LDHA showed a more evident correlation with immunotherapy resistance and was associated with tumor metastasis and TNM stage in NSCLC (Figure S1F and G, Supporting Information). Given that LDHA is involved in the synthesis of lactate, we hypothesized that lactate‐induced lactyl‐proteins or secreted small‐molecule metabolites might contribute to immunotherapy resistance in NSCLC.

Six fresh NSCLC tissues and paired normal tissues (>2 cm from the edge of the tumor) were selected (Figure S2A, Supporting Information). After eliminating the residual blood and connective tissue, a 3D mass spectrometry approach was employed to detect the total protein lactation modifications (Figure 1B). There were significant differences in Kla between the normal tissues and tumor tissues (Figure S2B, Supporting Information). We identified 2193 lysine lactylation (Kla) sites from 806 proteins (Figure 1C; Figure S2C and Table S1, Supporting Information), including 49 histone Kla sites (Figure S2D, Supporting Information) and 2144 non‐histone Kla sites. Upon conducting a quantitative comparison of the lactylome between tumor and adjacent lung tissues, a marked enrichment of Kla motifs and a prevailing upregulation, rather than downregulation, of Kla sites in the tumors was observed (Figure 1D and E). Significantly altered lactylated Kla sites were identified, including APOC2_K70 (Figure 1F). There was no difference between adenocarcinoma (AC) and squamous cell carcinoma (SCC) in terms of protein lactylation modification (Figure S3A and B, Supporting Information). However, the upregulated lactyl‐proteins were associated with many physiological and pathological signaling pathways in samples from patients treated with neoadjuvant therapy (Pembrolizumab+Platinum‐based chemotherapy) (Figure S3E and Table S2, Supporting Information), suggesting their potential role in NSCLC treatment.

Moreover, cellular compartment analysis showed that 39.58% of lactyl‐proteins resided in the cytoplasm (Figure 1G), which was possibly associated with higher cytoplasmic lactate content.^[^ ^16^ ^]^ Moreover, 35.41% of lactyl‐proteins were in the nucleus, exhibiting a higher ratio of upregulated lactyl‐proteins compared to downregulated ones in the tumor tissues (Figure 1G). Subsequent Kyoto Encyclopedia of Genes and Genomes (KEGG) pathway analysis showed the involvement of upregulated and downregulated lactyl‐proteins in various physiological and pathological signaling pathways (Figure 1H). Interestingly, no downregulated lactyl‐proteins were observed in the genetic information processing, such as translation and transcription. This could potentially be attributed to the rapid growth rate of tumors compared to that of the adjacent lung tissues. However, the number of downregulated lactyl‐proteins exceeded the number of upregulated proteins in lipid metabolism, implying the complexity of lipid metabolism regulation in tumor tissues (Figure 1H). Collectedly, our investigation revealed several non‐histone lactyl‐proteins with distinct subcellular localizations, linked to diverse signaling pathways.

Histone Kla is associated with poor prognosis in melanoma and other cancers.^[^ ^17^ ^]^ The abundance of non‐histone Kla prompted us to investigate its impact on NSCLC, western blotting revealed inconsistencies in histone (≈20 kDa) and non‐histone Kla levels (red arrow in Figure S4A, Supporting Information). Our quantitative analysis showed significant differences, with ≈25% of the proteins displaying notable variation (N = 40) (Figure S4B, Supporting Information). On further examining the correlation between total non‐histone Kla levels and tumor progression, a positive correlation was observed between the TNM stage, tumor metastasis, immunotherapy resistance, and tumor recurrence (Figure S4C–F). Similar trends were observed in breast cancers, with higher total non‐histone Kla levels linked to increased metastasis and decreased survival (Figure S4G–I, Supporting Information). Overall, our results suggest that non‐histone Kla may be an important independent factor in tumor metastasis and immune tolerance dynamics.

### 2.2 Lactate Promotes the Release of Extracellular FFAs from TG via Lactyl‐Proteins

**Figure 2:**
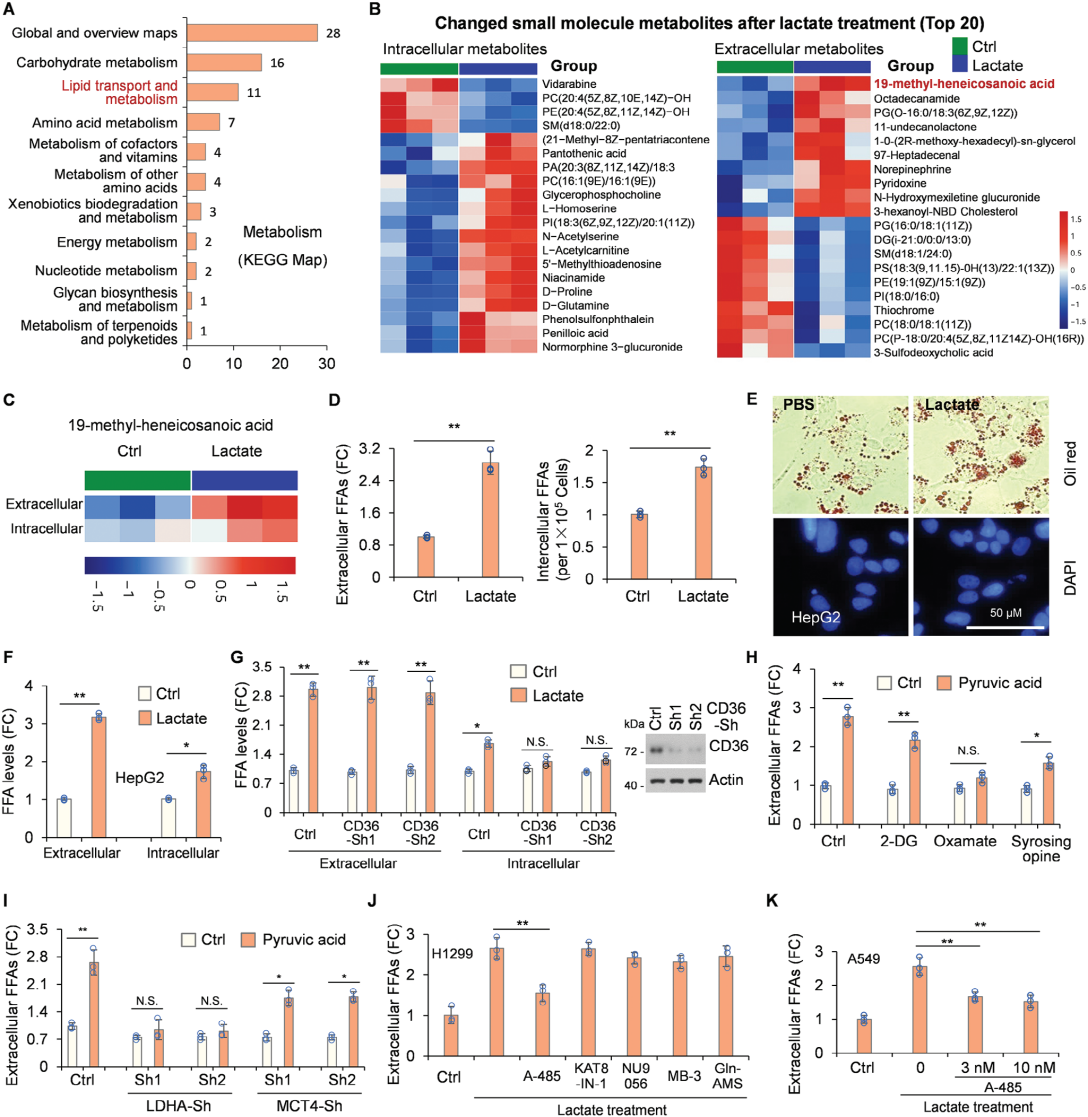
Lactate promotes the release of extracellular FFAs from TG via lactyl‐proteins. A) Global lactylome profiling showing the numbers of lactylated‐proteins in various pathways, as identified by KEGG map analysis in tumors. B) Top 20 intracellular and extracellular small molecule metabolites in tumor cells showing significant changes (*p* < 0.05) after 14 h lactate treatment, as analyzed by LC‐MS/MS. C) Change in intracellular and extracellular 19‐methyl‐heneicosanoic acid in tumor cells after lactate treatment. D–K) Analysis of intracellular or extracellular FFAs in the indicated tumor cells. Data are presented as mean ± SD from n ≥ 3 independent experiments, *p < 0.05, **p < 0.01, Student's t‐test. D) Analysis of FFAs in H1299 tumor cells after 14 h treatment with 20 mM lactate. (E and F) Analysis of intracellular or extracellular FFAs and oil red staining in HepG2 cells treated with 30 mM lactate for 24 h. Representative images show cells stained with oil red. G) H1299 cells were infected with either control shRNA, CD36‐Sh1, or Sh2 lentiviruses for 72 h, followed by another 14 h lactate treatment. CD36 knockdown was confirmed via Western blotting. H and I) Intracellular lactate promotes the increase of extracellular FFAs. H1299 cells were infected with control shRNA, LDHA‐Sh1, LDHA‐Sh2, MCT4‐Sh1, or MCT4‐Sh2 lentiviruses for 72 h, or treated with specific inhibitors for 12 h, followed by a 14 h pyruvic acid (5 mM) treatment. Extracellular FFAs were analyzed in the indicated cells. J and K) H1299 cells (J) and A549 cells (K) were treated with lactate (20 mM), A‐485[10 nM, (J); 3/10 nM, (K)], KAT8‐IN‐1 (300 µM), NU9056 (2 µM), Butyrolactone 3 (MB‐3, 300 µM), or Gln‐AMS (10 µM) for 14 h. Quantitative analysis of extracellular FFAs was conducted post‐treatment.

Global lactylome profiling revealed the levels of total non‐histone Kla in different metabolic pathways. Notably, a substantial abundance of lactylated non‐histone proteins was observed during carbohydrate metabolism and lipid transport and metabolism (**Figure** **2**A). A metabolomic study of the small molecules in lactate‐treated NSCLC cells (Figure S5A, Supporting Information) showed significant metabolic differences between the control and lactate‐treatment groups (Figure S5B, Supporting Information). The altered small‐molecule metabolites or signaling pathways (top 20) are shown in Figure 2B and Figure S5C (Supporting Information).

Remarkably, beyond the well‐documented molecules in the lactate metabolism pathway, we identified extracellular regulation of 19‐methyl‐heneicosanoic acid (ranking first among the extracellular metabolites) (Figure 2B). This molecule is one of the significant forms of FFAs, and its regulation is independent of the lactate metabolism pathway.^[^ ^18^ ^]^ Furthermore, FFA levels was also upregulated in intracellular compartments (Figure 2C). To confirm whether lactate regulates lipid metabolism, we evaluated extracellular and intracellular FFA levels levels in tumor cells following lactate treatment. Both extracellular and intracellular FFA levels increased significantly upon lactate treatment of H1299 cells (Figure 2D). Since excess FFAs are used to synthesize triglycerides (TG) in hepatocytes, hepatoma HepG2 cells, in which TG staining with Oil Red is visible, were selected to verify the influence of lactate on lipid metabolism. TG, as well as extracellular and intracellular FFAs, increased, supporting our hypothesis (Figure 2E and F).

FFA accumulation involves TG hydrolysis in the extracellular space and de novo fatty acid synthesis in cells.^[^ ^19^, ^20^, ^21^ ^]^ Excess FFAs are transported into cells by CD36 to maintain cellular metabolism.^[^ ^22^ ^]^ To determine whether the increase in FFAs was due to intracellular or extracellular sources, CD36 was knocked down. We found that extracellular FFA levels remained elevated, whereas intracellular FFAs no longer displayed significant increases in CD36‐Sh cells, indicating that the augmented intracellular FFA levels primarily resulted from enhanced FFA uptake rather than intracellular FFA synthesis (Figure 2G).

To explore whether extracellular or intracellular lactate promotes extracellular lipolysis, we used 2‐DG, oxamate, and syrosingopine, which inhibit the conversion of glucose to pyruvate, pyruvate to lactate, and lactate export from tumor cells, respectively. Under oxamate treatment, the lactate‐induced FFAs increase was no longer apparent, whereas 2‐DG and syrosingopine exerted no effect at high pyruvate concentrations (Figure 2H). These results indicated that intracellular lactate was sufficient to induce an increase in extracellular FFA levels. Consistently, lactate‐induced FFA elevation was attenuated in LDHA‐Sh cells, but not in MCT4‐Sh cells (Figure 2I; Figure S5D, Supporting Information). Collectively, these observations suggested a causative relationship between intracellular lactate levels and the production of extracellular FFAs.

To ascertain whether the lactate‐induced increase in extracellular FFAs is mediated through lactyl‐proteins, we chose A‐485 (an inhibitor targeting lactyltransferases p300/CBP),^[^ ^23^ ^]^ KAT8‐IN‐1 (targeting KAT8, KAT2B, and KAT3B),^[^ ^24^ ^]^ NU9056 (targeting KAT5),^[^ ^25^ ^]^ MB‐3 (targeting KAT2A),^[^ ^26^ ^]^ and Gln‐AMS (targeting AARS1 and AARS2),^[^ ^27^ ^]^ and found that only A‐485 inhibited the increase of extracellular FFAs in H1299 and A549 cells, indicating that lactyl‐proteins are needed in lactate‐induced FFAs (Figure 2J,K). These data suggest that lactate‐induced extracellular FFAs release from TG is dependent on lactyl‐proteins.

### 2.3 Lactate Enhances APOC2 Lactylation at K70 Site and Subsequent Extracellular Lipolysis

**Figure 3:**
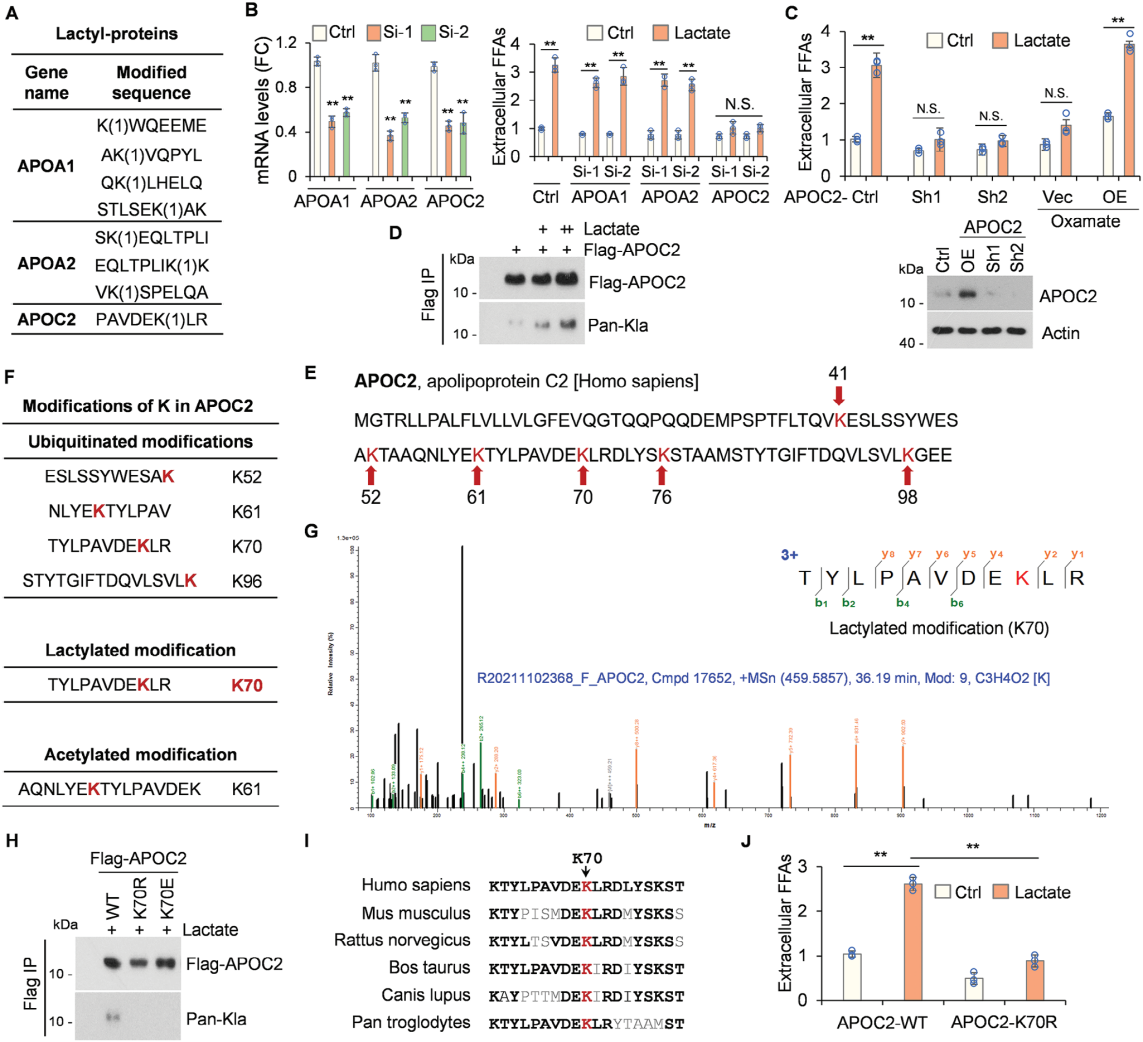
Lactate enhances APOC2 lactylation at K70 site and subsequent extracellular lipolysis. A) Table presents the lactyl‐proteins involved in FFA synthesis related to lipid transport and metabolism, identified through 3D mass spectrometry targeting total lactyl‐proteins. B) H1299 cells were transfected with control siRNA, APOA1‐Si‐1, APOA1‐Si‐2, APOA2‐Si‐1, APOA2‐Si‐2, APOC2‐Si‐1, or APOC2‐Si‐2 for 48 h, followed by lactate treatment for 14 h. Q‐PCR was employed to detect the knockdown of indicated genes. Extracellular FFAs in the indicated samples were analyzed. C) H1299 cells were treated with control shRNA, APOC2‐Sh1, or APOC2‐Sh2 lentiviruses for 72 h, or transfected with APOC2‐overexpression for 12 h, followed by 14 h lactate or oxamate treatment. Extracellular FFAs in the indicated samples were analyzed. APOC2 protein level was detected using Western blotting. D) 293T cells were transfected with the indicated Flag‐APOC2 plasmids, followed by immunoprecipitation with FLAG‐M2 beads. The precipitated proteins were analyzed using Western blot with Flag or pan‐Kla antibodies. E) Six lysine (K) residues in APOC2 proteins are highlighted in red. F–H) 293T cells were transfected with Flag‐APOC2 plasmids for 12 h and treated with lactated for another 14 h, followed by immunoprecipitation with FLAG‐M2 beads. The samples were analyzed using SDS‐PAGE and Coomassie brilliant blue staining. The APOC2 protein band was cut out for mass spectrometry analysis. F) Table showing all K modifications in APOC2, including four ubiquitinated, one lactylated, and one acetylated modification. G) B‐y ion matching diagrams of APOC2‐K70 lactylation site was displayed. H) 293T cells were transfected with Flag‐APOC2 ‐WT, ‐K70R, or ‐K70E plasmids, followed by immunoprecipitation with FLAG‐M2 beads. The precipitated proteins were analyzed by western blot using Flag or pan‐Kla antibodies. I) APOC2‐K70 sequence alignment from the indicated species is shown. J) H1299 cells were transfected with APOC2 ‐WT or ‐K70R plasmids for 12 h, then treated with lactate for another 14 h. Extracellular FFAs were analyzed in the indicated samples. The statistical data in this figure represents the mean ± SD from n ≥ 3 independent experiments, *p < 0.05, **p < 0.01, N.S. = no significant, Student's t‐test.

To unravel the mechanistic basis of FFAs upregulation by lactate, we analyzed the lactyl‐non‐histone proteins involved in lipid transport and metabolism (**Figure** **3**A; Figure S6A, Supporting Information). Apolipoproteins, the major lipoproteins involved in lipid transport, supply lipids that serve as vital energy sources for high levels of cancer cell proliferation and invasion.^[^ ^28^, ^29^, ^30^, ^31^, ^32^ ^]^ We found that only APOC2‐Si inhibited the lactate‐induced increase in extracellular FFAs, but not APOA1‐Si or APOA2‐Si (Figure 3B). Moreover, APOC2 was the only upregulated lactyl‐protein in tumors compared to adjacent normal tissues (Figure 1F). Apolipoprotein C2 (APOC2) forms a complex with the enzyme lipoprotein lipase (LPL) to facilitate the hydrolysis of TG, thereby generating FFAs for cellular metabolism.^[^ ^33^, ^34^ ^]^ LPL is markedly upregulated in NSCLC, which significantly reduces the survival time of these patients.^[^ ^35^ ^]^ Extracellular FFA levels were not induced when APOC2 was knocked down, and were increased after APOC2 overexpression even when oxamate was used to inhibit lactate levels (Figure 3C). These data suggest that lactate‐induced extracellular FFAs release from TG is dependent on APOC2.

To confirm the lactylation of APOC2 in response to lactate, we treated cells with lactate and observed a concentration‐dependent increase in lactyl‐APOC2 levels (Figure 3D). Analysis of the amino acid sequence of APOC2 identified six lysine (K) residues: K41, K52, K61, K70, K76, and K98 (Figure 3E). All lactylation sites in APOC2 was indentified by overexpressing APOC2 in 293T cells, followed by mass spectrometry analysis (Figure 3F). One site for lactylation, four sites for ubiquitination, and one site for acetylation were identified. The b‐y ion matching diagram showed that APOC2 was lactylated at K70 and ubiquitinated at K52, K61, K70, or K96 (Figure 3G; Figure S6B–F, Supporting Information). This observation is consistent with our lactylome profiling analysis, which identified K70 as a potential lactylation site (Figure 3A).

Subsequently, a lactylation‐defective mutant (K to R) and another mutant (K to E) at the K70 position were generated.^[^ ^9^ ^]^ It was observed that the pan‐Kla antibody could not recognize APOC2‐K70 mutants under lactate treatment, but recognized APOC2‐wild‐type (WT) (Figure 3H). These data indicated that K70 is the only lactylation site for APOC2. Notably, K70 is a highly conserved amino acid residue in APOC2 across various species (Figure 3I). In addition, lactate facilitated the release of extracellular FFAs in APOC2‐WT overexpression cells, but not in APOC2‐K70R overexpression cells (Figure 3J). These results suggested that lactate enhanced APOC2 lactylation at K70, leading to extracellular lipolysis.

### 2.4 P300 Promotes APOC2‐K70 Lactylation and Subsequent APOC2 Accumulation

**Figure 4:**
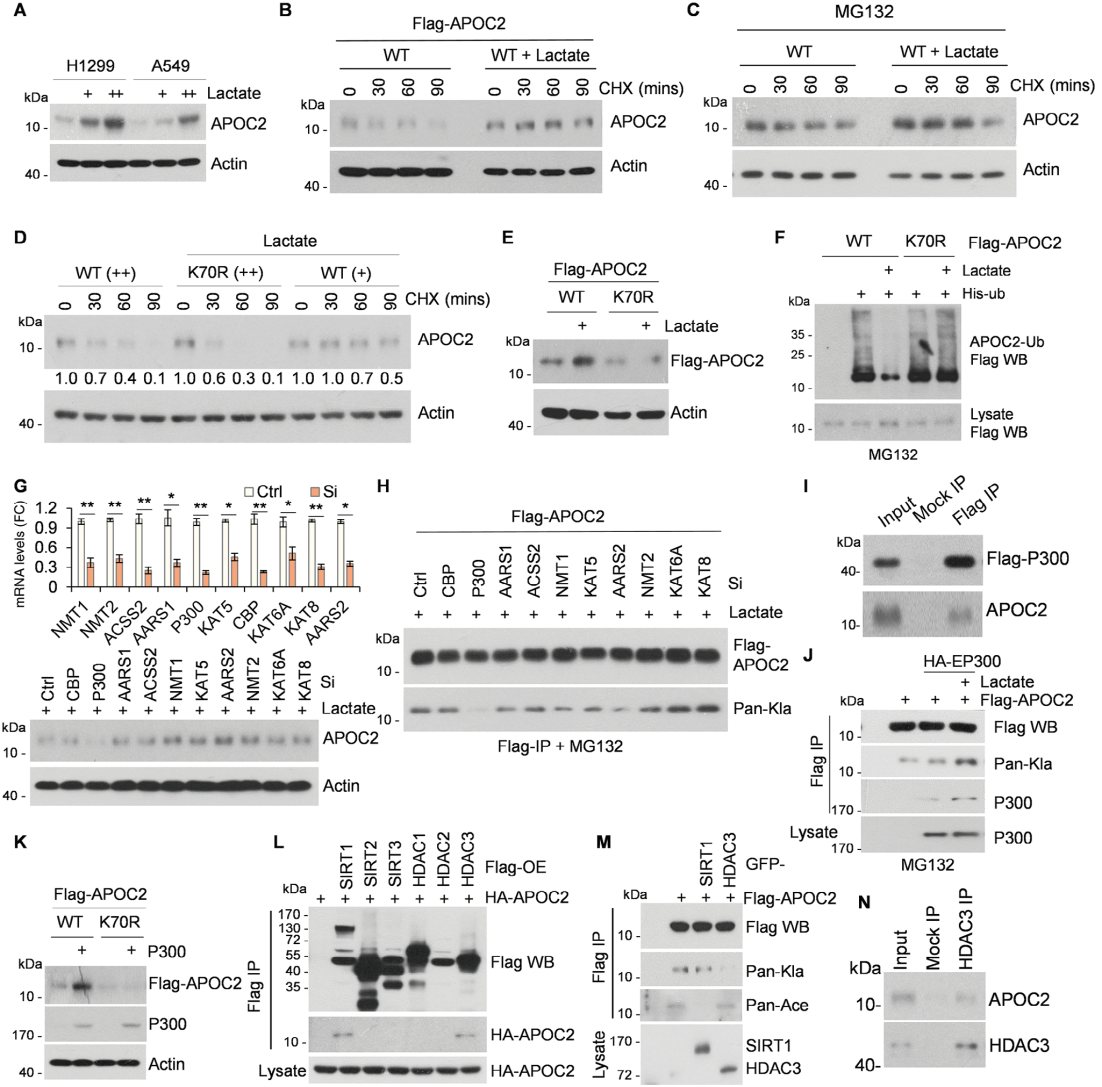
P300 promotes APOC2‐K70 lactylation and subsequent APOC2 accumulation. A–D) Lactate promotes the accumulation of APOC2 protein by enhancing its stability. H1299, A549, or 293T cells were treated with lactate (5/20 mM) for 14 h. Endogenous or exogenous APOC2 and β‐actin were detected by APOC2, Flag, or β‐actin antibodies. B and C) 293T cells were transfected with Vector or Flag‐APOC2 for 24 h, and then treated with the translation inhibitors cycloheximide (CHX, 50 µg mL^−1^) or MG132 (25 µM, 4 h) for the indicated durations. D and E) H1299 cells were transfected with Flag‐APOC2‐WT or ‐K70R for 12 h, and then treated with lactate (20 mM) for another 14 h. Cell lysates were subjected to Western blot using FLAG or β‐actin antibodies. D) H1299 cells were transfected with different amounts of Flag‐APOC2‐WT (+/++) or ‐K70R (++) plasmids. Cells were treated with CHX (50 µg mL^−1^) for the indicated durations. The relative APOC2 band intensities were quantified using densitometry and presented in relation to 0 h controls. F) Lactate inhibits APOC2 ubiquitination. 293T cells were transfected with his‐ubiquitin, Flag‐APOC2‐WT, or ‐K70R plasmids. Ubiquitinated proteins were precipitated using Ni‐NTA beads. G–K) P300 binds to APOC2, and promotes APOC2‐K70 lactylation after lactate treatment. Immunoprecipitation analysis was conducted using Flag‐M2 beads. Western blot was performed for endogenous APOC2, exogenous APOC2, APOC2^K70‐lac^, or P300 using anti‐APOC2, ‐Flag, pan‐Kla, or HA antibodies. G) qRT‐PCR analysis of mRNA levels of potential APOC2‐related lactyltransferases in control and ‐siRNA H1299 cells (upper panel). Western blotting analysis showed endogenous APOC2 and β‐actin protein levels. H) 293T cells were transfected with Flag‐APOC2, Ctrl‐Si, or indicated siRNA for 48 h, and then treated with MG132 (25 µM, 4 h) before cells lysis. I) H1299 cells transfected with Flag‐P300 were precipitated using Flag‐M2 beads or IgG (mock IP), and co‐precipitated APOC2 was detected by western blot. J) Flag‐APOC2 and HA‐P300 plasmids were co‐transfected into 293T cells, followed by Flag immunoprecipitation. MG132 (25 µM, 4 h) were added before cells lysis. K) H1299 cells were transfected with Flag‐P300 or APOC2‐WT, ‐K70R or −70E plasmids for 12 h, and then treated with lactate (20 mM) for another 14 h. L–N) HDAC3 delactylates APOC2‐K70 lactylation. Cell lysates were immunoprecipitated using Flag‐M2 beads and western blot was performed for delactylases, APOC2 or lactyl‐APOC2 using anti‐Flag, HA, GFP, pan‐Kla, or pan‐Ace antibodies. L) SIRT1 and HDAC3 bound to APOC2. HA‐APOC2 and Flag‐SIRT1, ‐SIRT2, ‐SIRT3, ‐HDAC1, ‐HADC2, or ‐HDAC3 plasmids were co‐transfected into 293T cells followed by immunoprecipitation. M) GFP‐SIRT1, ‐HDAC3, or Flag‐APOC2 were co‐transfected into 293T cells followed by Flag immunoprecipitation. N) Endogenous HDAC3 in H1299 cells was precipitated using IgG (mock IP) or HDAC3 antibody. Co‐precipitated APOC2 was detected by western blot.

Protein modifications can affect protein activity or stability. To confirm the function of APOC2‐K70 lactylation, we treated H1299 and A549 cells with lactate and observed a concentration‐dependent increase in APOC2 protein level, without affecting its transcription (**Figure** **4**A; Figure S7A, Supporting Information). Following cycloheximide (CHX) treatment, lactate indeed stabilized APOC2 protein (Figure 4B), which was disrupted by MG132, a proteasome inhibitor (Figure 4C). Moreover, even in the presence of lactate, lactylation‐defective mutant APOC2‐K70R exhibited a faster degradation rate than APOC2 ‐WT with or without lactate treatment (Figure 4D). Lactate treatment did not induce an increase in the protein levels of APOC2‐K70R, but caused an increase in those of the ‐WT (Figure 4E). The ubiquitination assay confirmed that lactate treatment reduced the ubiquitination of APOC2‐WT, but not of lactylation‐defective mutant APOC2‐K70R (Figure 4F). These results indicate that lactylation of APOC2 at K70 stabilizes the protein levels by inhibiting its ubiquitination.

To explore the potential lactyltransferases or modifying enzymes of APOC2‐K70, siRNA knockdown of ten candidate proteins was performed.^[^ ^10^ ^]^ It was observed that abrogation of P300 prevented the increase in APOC2 protein levels upon lactate treatment (Figure 4G). Specifically, only the knockdown of P300 led to a significant decrease in APOC2‐K70 lactylation (Figure 4H). Combined with the findings shown in Figure 2G, we propose that P300 is the primary lactylation enzyme of APOC2‐K70. To eliminate the effects of differences in protein stability of APOC2 under different conditions, MG132 was added to the cells before cell lysis. Moreover, the interaction of P300 with endogenous APOC2 (Figure 4I) was significantly enhanced upon lactate stimulation, leading to an increase in K70 lactylation (Figure 4J). P300 overexpression increase the protein levels of APOC2‐WT, but not those of APOC2‐K70R (Figure 4K). Overall, P300, the primary lactyltransferase of APOC2‐K70, regulates APOC2 protein stability.

SIRT1‐3 and HDAC1‐3 have been reported to be major delactylases.^[^ ^36^, ^37^, ^38^ ^]^ Co‐transfection of these enzymes with APOC2 showed that SIRT1 and HDAC3 primarily interacted with APOC2 (Figure 4L). Co‐expression with HDAC3 decreased lactylation levels without affecting acetylation, whereas co‐expression with SIRT1 decreased acetylation levels without affecting lactylation (Figure 4M). Endogenous HDAC3‐APOC2 interaction was also detected (Figure 4N). Overexpression or knockdown of HDAC3 significantly affected the total protein level of APOC2 (Figure S7B, Supporting Information). Moreover, His‐HDAC3 purified from *Escherichi coli* was capable of delactylating APOC2 in vitro (Figure S7C, Supporting Information). These findings support the role of HDAC3 as a delactylase of APOC2‐K70. Additionally, a negative correlation between HDAC3 and APOC2 protein levels was observed in NSCLC and gastric cancer (GC) (Figure S7D and E, Supporting Information). Given the inherent duality of lactylation enzymes as acetylation catalysts and the identification of one potential acetylation site on APOC2, further exploration is required to uncover the interplay between APOC2's lactylation and acetylation (mass spectrometry data, Figure 3F). SIRT1, but not HDAC3, possesses the ability to deacetylate APOC2 (Figure 4M). Nevertheless, the acetylation‐deficient mutant APOC2‐K61R did not affect the protein stability of APOC2 (Figure S7F, Supporting Information).

### 2.5 Lactyl‐APOC2‐K70 Promotes Extracellular Lipolysis and Tumor Metastasis

**Figure 5:**
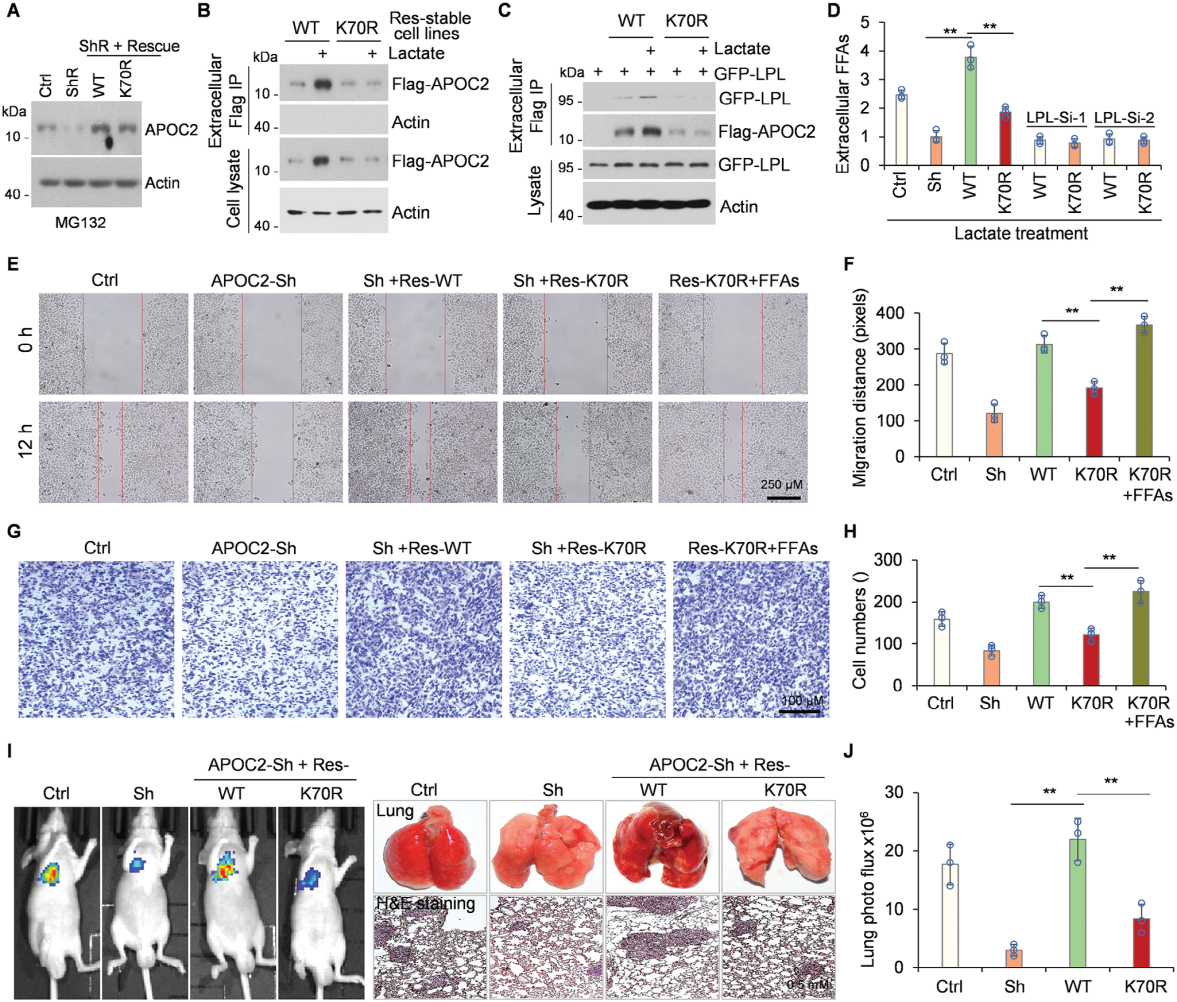
Lactyl‐APOC2‐K70 promotes FFAs release and tumor metastasis. A) Establishment of stable cell lines expressing Flag‐tagged APOC2‐WT or ‐K70R mutants from H1299 cells. MG132 (25 µM, 4 h) were added before cells lysis. Cell lysates were immunoblotted with antibodies against APOC2 with β‐actin serving as the internal standard. B and C) Indicated stable cell lines were treated with lactate for 14 h, and the culture medium from the samples was immunoprecipitated using FLAG‐M2 beads. The immunoprecipitation proteins and cell lysates were analyzed by western blot using anti‐ Flag, GFP, or β‐actin antibodies. C) GFP‐LPL plasmids were co‐transfected into indicated stable cell lines for 30 h. D) Extracellular FFAs were analyzed in the indicated cells. Stable cell lines were transfected with LPL‐Si‐1 or LPL‐Si‐2 for 36 h, and then were treated with lactate for 14 h. *p < 0.05, **p < 0.01, Student's t‐test. E and F) A wound‐healing assay was performed on the indicated stable cell lines with or without FFAs (OA:PA = 2:1, 0.5 mM) treatment. Representative images (E) and quantification of metastasis (F) were presented. G and H) Boyden chamber Matrigel invasion assays were conducted on the indicated stable cells with or without FFAs (OA:PA = 2:1, 0.5 mM, 24 h) treatment. Representative images (G) and Quantification of metastasis (H) were showed. I and J) 2×10^5^ indicated cells carrying luciferase expression system were administrated into mice by tail vein injection for 42 days. The luciferase activity was assessed using a noninvasive imaging system (xenogen). I) Representative images were captured after subcutaneously injecting 150 mg kg^−1^ D‐luciferin substrate in PBS to anesthetized mice (left). The lung organs from tumor‐bearing mice were fixed and processed with H&E staining of section (right). J) Quantification of metastasis to the lungs via luciferase expression was measured in photons per sec (mean ± SD; n = 6).

APOC2 together with LPL in TME hydrolyzes TG, thereby generating FFAs for cellular metabolism.^[^ ^33^, ^34^ ^]^ To examine the function of APOC2‐K70 lactylation in regulating FFAs release, a systematic investigation was undertaken using engineered cell lines carrying APOC2 knockdown and subsequent rescue of WT and lactylation‐deficient K70R (**Figure** **5**A). To eliminate the effect of differences in protein stability of APOC2‐WT or mutants, we used MG132, a proteasome inhibitor before cells lysis (Figure 5A). Discernible reductions in APOC2 protein secretion were observed in APOC2‐K70R cells compared to APOC2‐WT cells (Figure 5B). Moreover, lactate treatment led to an increased interaction of WT APOC2, but not of mutant APOC2‐K70R, with LPL (Figure 5C). Extracellular FFA levels were significantly reduced in APOC2‐Sh, APOC2‐K70R, LPL‐Si‐1, and LPL‐Si‐2 cells, indicating that this process required lactyl‐APOC2 and LPL (Figure 5D; Figure S7G, Supporting Information). These results indicated that lactyl‐APOC2‐K70 promoted FFAs release from TG.

Numerous studies have demonstrated a correlation between FFA levels and tumor metastasis.^[^ ^39^, ^40^ ^]^ Therefore, the role of lactyl‐APOC2 in tumor metastasis was investigated. As shown in Figure 5E–H, APOC2‐WT promoted tumor migration, while ‐K70R did not. Moreover, the addition of FFAs increased the migration of APOC2‐K70R tumor cells in both the cell scratch and transwell assays. The in vivo data also showed that APOC2‐WT promoted pulmonary metastasis, unlike APOC2‐K70R (Figure 5I,J). This suggests that lactyl‐APOC2 promotes pulmonary metastasis.

### 2.6 Lactyl‐APOC2‐K70 Promotes Tregs Augmentation and Immunotherapy Resistance

**Figure 6:**
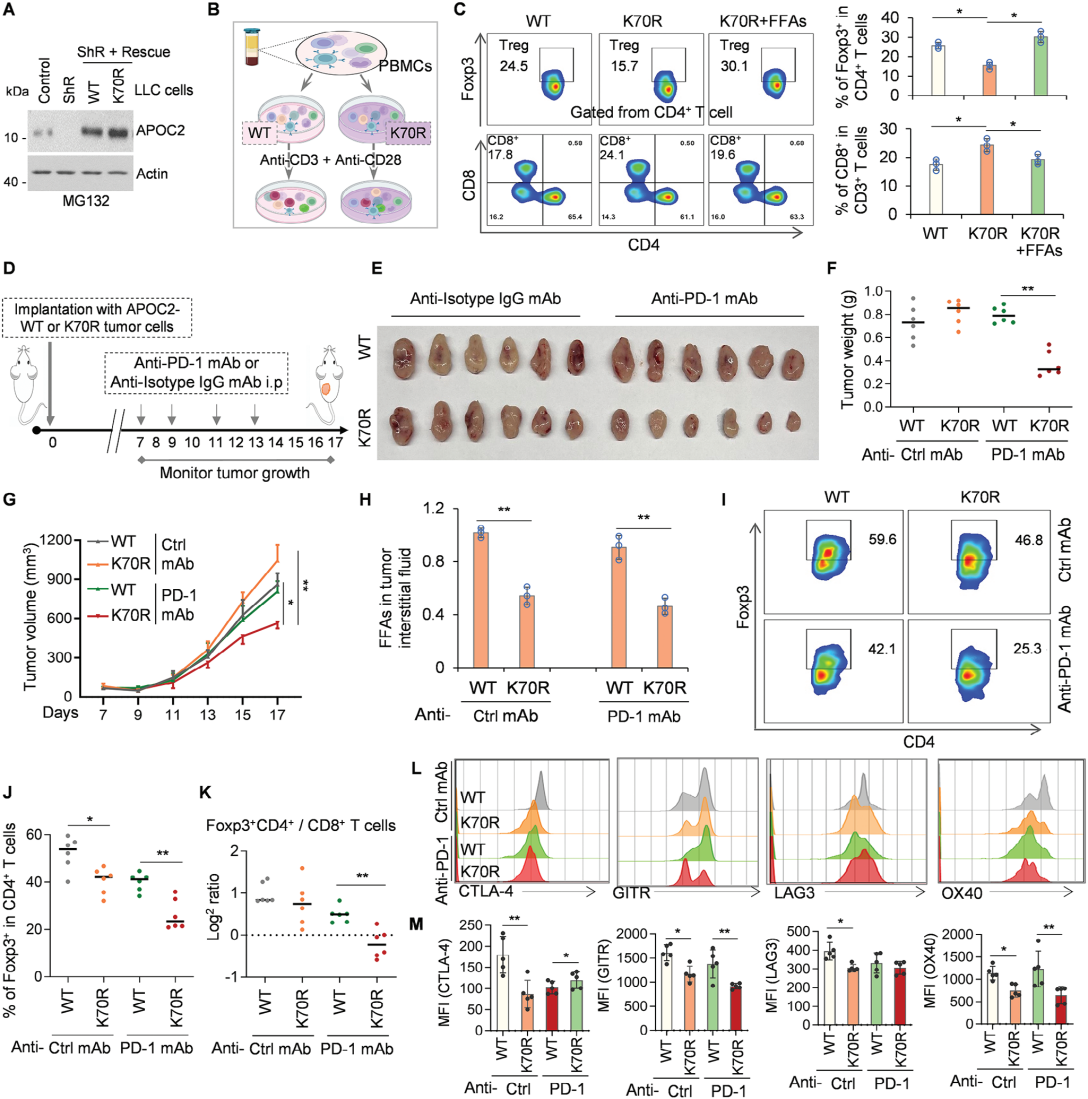
Lactyl‐APOC2‐K70 promotes Tregs augmentation and immunotherapy resistance. A) Establishment of stable LLC cell lines expressing Flag‐tagged APOC2‐WT or ‐K70R (K67 in murine) mutants were based on APOC2‐knockdown cells. MG132 (25 µM, 4 h) were added before cells lysis. Cell lysates were immunoblotted with antibodies against APOC2 and β‐actin. B and C) Schematic of PBMCs co‐cultured with the tumor cells, followed by flow cytometry (FCM). PBMCs were successfully separated and stimulated with anti‐CD3 mAb and anti‐CD28 mAb for 72 h, then co‐cultured with APOC2‐WT or ‐K70R tumor cell lines, or supplemented with FFAs (OA:PA = 2:1, 0.5 mM). The mixtures were collected for subsequent FCM. (C) The frequency of Tregs (Foxp3^+^CD4^+^ T) cells and CD8^+^ T cells in CD3^+^ T cells in mixtures were examined. (D‐K) Lactyl‐APOC2‐K70 leads to resistance after anti‐PD‐1 mAb treatment. D) Schematic of the short‐term in vivo treatment of mice with anti‐PD‐1 antibodies in APOC2‐WT or ‐K70R xenograft tumors. Each group was treated either with isotype control (Vehicle) or anti‐PD‐1 antibody on days 7, 9, 11, and 13 (four doses), and then on day 17 mice were sacrificed for analysis. E) Xenograft tumors borne by APOC2‐WT or ‐K70R (‐K67R in murine) cells were collected and photographed (n  =  6). F and G) Quantification of average tumor weight and volume. Six tumors were included in each group. H) FFAs in tumor interstitial fluid was analyzed in indicated tumors. I–M) TIL prepared from tumor tissue samples were subjected to FCM. I–K) The frequencies of Foxp3^+^CD4^+^ T cells, the ratio of Foxp3^+^CD4^+^ to CD8^+^ T cells in the TME were examined with FCM. Representative contour plots I) and summaries J and K) are shown. L and M) The expression of CTLA‐4, GITR, LAG‐3, and OX‐40 by Foxp3^+^CD4^+^ T cells in the TME was examined with FCM. Representative histograms and MFI summaries are shown. Data represent the mean ± SD of three times of independent experiments. N.S. = not significant, *p < 0.05, **p < 0.01.

Given that FFAs promotes metabolic advantages in regulatory T Cells,^[^ ^7^, ^8^ ^]^ we investigated the role of lactyl‐APOC2 in prolonging the survival of Tregs. To assess the immunological effects of APOC2 K70 mutations on tumor immune microenvironment of lung cancer, we established APOC2^Sh^ rescue ‐WT and ‐K70R murine lung cancer cell lines (LLC cells) (**Figure** **6**A). Peripheral blood mononuclear cells (PBMCs)^[^ ^41^ ^]^ from healthy individuals were co‐cultured with APOPC2‐WT or ‐K70R cancer cells (Figure 6B). After coculturing with APOPC2‐WT cells, flow cytometry analysis showed a significant increase in the frequency of CD4^+^ FOXP3^+^ Tregs and a decrease in the frequency of CD8^+^ effector T cells compared to co‐culturing with APOPC2‐K70R cells (Figure 6C). Additionally, the addition of FFAs led to an increase in the frequency of CD4^+^ FOXP3^+^ Tregs co‐culturing with APOPC2‐K70R in vitro, indicating that lacty‐APOC2 promoting Tregs augmentation was dependent on FFAs release (Figure 6C).

We investigated the in vivo function of lactyl‐APOC2‐K70 on Tregs expansion and the efficacy of anti‐PD‐1 therapy in an allograft mouse model. Anti‐PD‐1 mAb or anti‐isotype IgG mAb was intraperitoneally injected into mice carrying APOC2‐WT or −K70R LLC cells (Figure 6D). We found that anti‐PD‐1 mAb failed to inhibit the growth of tumors with APOC2‐WT compared to that of tumors with APOC2‐K70R (Figure 6E–G). FFAs in the tumor interstitial fluid were also decreased in APOC2‐K70R tumors compared to APOC2‐WT tumors, indicating that lactyl‐APOC2‐K70 promotes FFAs release (Figure 6H). Tregs effectively utilizes FFAs for survival, whereas conventional CD4^+^ T cells and CD8^+^ T cells are likely to depend on glucose consumption.^[^ ^42^ ^]^ The frequency of Treg cells increased in tumors with APOC2 ‐WT, with decreased frequency of CD8^+^ T cells, resulting in a higher ratio of Treg cells to CD8^+^ T cells, compared to tumors with APOC2‐K70R (Figure 6I–K). The frequency of active markers in Tregs (CTLA‐4, GITR, LAG‐3, and OX‐40) was higher in tumors with APOC2‐WT than in those with APOC2‐K70R (Figure 6L,M). In addition, the frequencies of tumor necrosis factor (TNF)‐α^+^interferon (IFN)‐γ^+^, CD62L^‐^CD44^+^, and CD69^+^ tumor‐infiltrating CD8^+^ T cells were increased by anti‐PD‐1 mAb in tumors with APOC2‐K70R (Figure S8A–D, Supporting Information). These results demonstrate that the lactylation of APOC2 at K70 leads to immunotherapy resistance in NSCLC by promoting the release of FFAs in vitro and in vivo.

### 2.7 Lactyl‐APOC2 is Associated with Tumor Progression and Immunotherapy Resistance in NSCLC Samples

**Figure 7:**
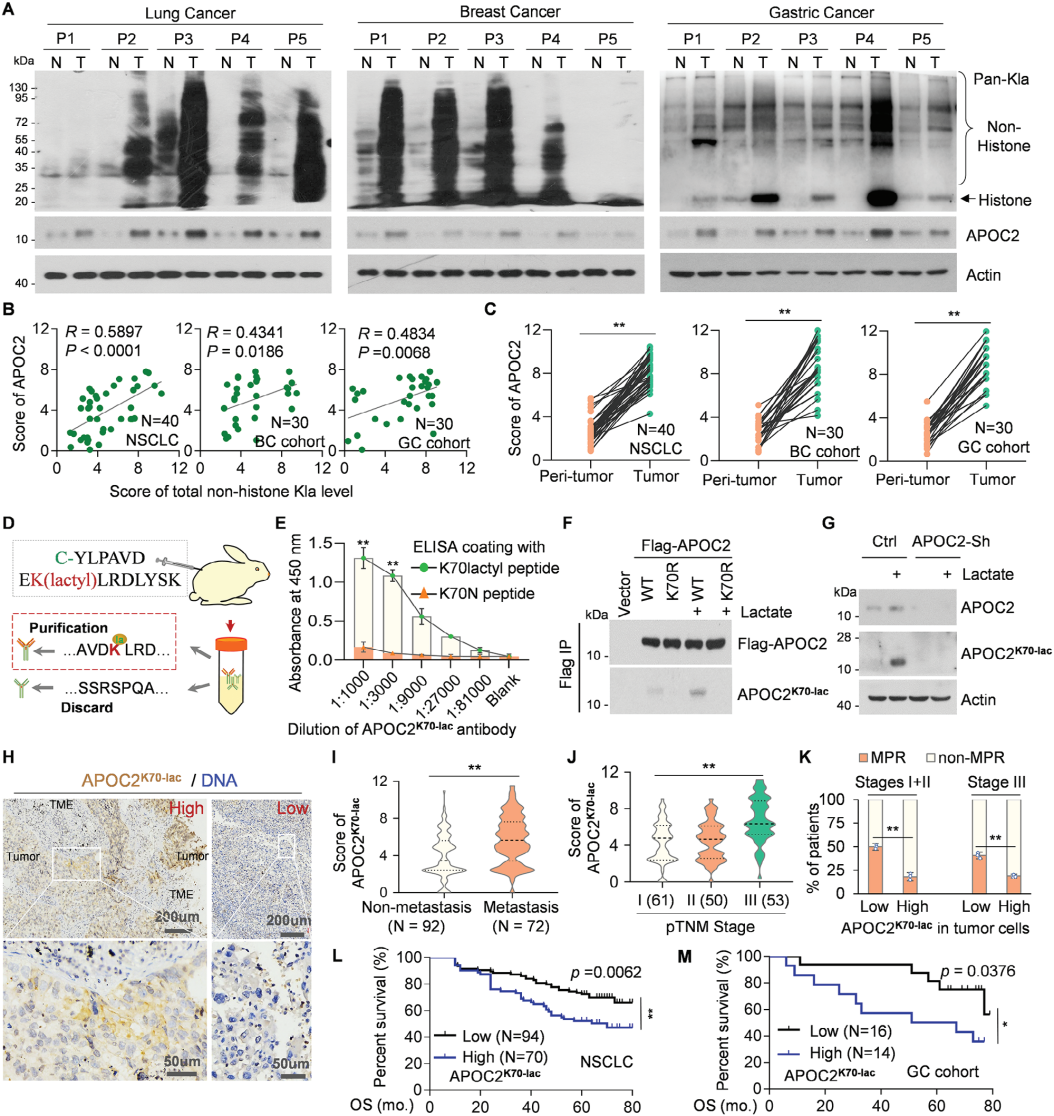
Lactyl‐APOC2 is associated with tumor progression and immunotherapy resistance in NSCLC samples. A and B) APOC2 protein levels is significantly related to the total non‐histone Kla level. A) Representative western blot shows the levels of total non‐histone Kla and APOC2 protein using pan‐Kla, APOC2, and β‐actin antibodies in NSCLC, breast cancers, gastric carcinoma (T), or paired adjacent tissues (N). B) Correlation between total non‐histone Kla level and APOC2 protein level in the indicated tumors. C) Paired line scatter plot shows APOC2 protein level in pairs of indicated tumors (green dot) and adjacent normal tissues (orange dot). D) Production process of anti lac‐APOC2‐K70 antibody (APOC2^K70‐lac^ Ab). E) ELISA analysis of APOC2^K70‐lac^ Ab is performed by incubating with lactyl‐peptide or N‐peptide coated wells. F) Identification of APOC2^K70‐lac^ Ab. 293T cells were transfected with wildtype or mutant forms of Flag‐APOC2 for 12 h, treated with lactate for another 14 h, and then immunoprecipitated with Flag‐M2 beads. The samples were analyzed by western blot using the antibody against APOC2^K70‐lac^ or Flag. G) Ctrl or APOC2‐Sh cells were treated with lactate for 14 h, followed by western blot using the antibody against APOC2, APOC2^K70‐lac^, or β‐actin. H–N) The APOC2^K70‐lac^ Level in 164 paired samples of NSCLC (H–M) or 30 samples of GC (N) were calculated on a scale of 1–12 points according to the staining of IHC in a double‐blinded manner. Scores below 6 are labeled as “low”, otherwise they were labeled as “high”. H) Representative immunohistochemical staining of APOC2^K70‐lac^ in NSCLC containing pairs of tumor and adjacent normal tissues. I–L) Correlation between Lactyl‐APOC2‐K70 level and patients with tumor metastasis (I), different stages (J), or major pathologic response (MPR) (K). L and M) Kaplan–Meier plot of Overall Survival of patients with NSCLC or GC cohorts stratified by low or high APOC2‐K70 levels. *p < 0.05, **p < 0.01 by Student's t‐test.

To further assess the clinical importance of APOC2‐K70 lactylation levels, we collected data from another 30 breast cancers (BC cohort) and 30 gastric cancers (GC cohort). Western blotting was performed to assess the levels of total non‐histone (Kla) and APOC2 proteins in the clinical specimens (**Figure** **7**A). A significant correlation between APOC2 protein levels and total non‐histone Kla was observed, with substantially higher APOC2 protein levels evident in tumor samples than in adjacent normal tissues (Figure 7A–C). This observation underscores the association between APOC2 protein levels and the aggregation of non‐histone Kla. Subsequently, we produced a specific antibody against APOC2 lactyl‐K70 using the C‐YLPAVDEK(lactyl)LRDLYSK peptide (Figure 7D), and confirmed its effectiveness and specificity (Figure 7E,F). This purified APOC2 lactyl‐K70 antibody accurately detected endogenous APOC2‐K70 lactylation, exhibiting minimal nonspecific bands under lactate treatment and negligible signals in APOC2‐Sh cells (Figure 7G). These results confirmed the reliability and specificity of the APOC2 lactyl‐K70 antibody.

We used this antibody to explore the relationship between APOC2‐K70 lactylation and tumor metastasis or immunotherapy resistance via immunohistochemistry (IHC) in clinical samples. We discovered that APOC2‐K70 lactylation was primarily localized in tumor cells and the extracellular space but not in immune cells or CAFs (Figure 7H). Furthermore, it was significantly associated with tumor metastasis (Figure 7I). Further analysis revealed that APOC2 protein levels were higher in stage III patients than in stage I or II patients (Figure 7J). Additionally, lactyl‐APOC2‐K70 levels were significantly elevated in NSCLC clinical samples that exhibited resistance to immunotherapy (Figure 7K). Moreover, lactyl‐APOC2‐K70 level was negatively correlated with overall survival (OS) in NCSLC and GC patients (Figure 7L,M). These results indicate that lactyl‐APOC2‐K70 is involved in tumor progression and immunotherapy resistance.

### 2.8 Anti‐APOC2^K70‐lac^ Ab Enhances Sensitivity to Anti‐PD‐1 Treatment by Inhibiting Lactyl‐APOC2

Subsequently, a significant elevation in Tregs cells (CD4^+^ FOXP3^+^) and a reduction in effector cells (CD8^+^/CD19^+^) in the tumor tissues of NSCLC clinical specimens from 40 patients undergoing combined chemotherapy and immunotherapy was observed (Figure S9A–C, Supporting Information). Lactyl‐APOC2‐K70 induced Tregs expansion and led to immunotherapy resistance (Figure 6), and a correlation between lactyl‐APOC2‐K70 and therapy resistance emerged in these tissues (Figure 7). Thus, we hypothesized that the inhibition of lactyl‐APOC2‐K70 by APOC2 lactyl‐K70 antibody or inhibition of lactate by FX11 (an LDHA inhibitor)^[^ ^43^ ^]^ might significantly promote immunotherapy in NSCLC.

**Figure 8:**
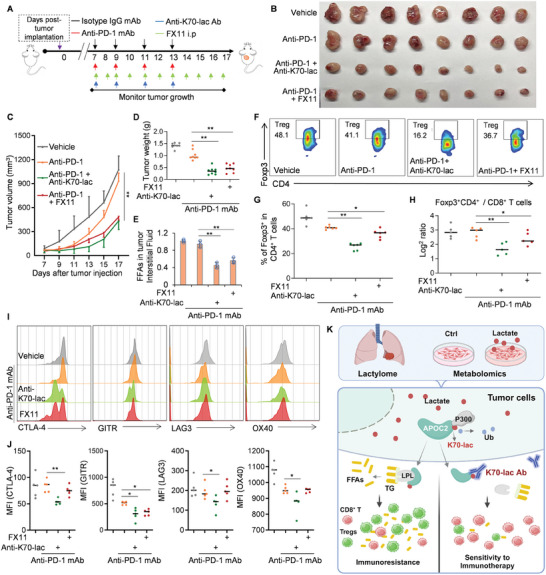
APOC2^K70^ ^‐lac^ antibody enhances sensitivity to anti‐PD‐1 treatment. A) Schematic of the short‐term in vivo treatment of mice with anti‐Isotype IgG or anti‐PD‐1 or anti‐APOC2^lac^ + anti‐PD‐1 or FX11 + anti‐PD‐1 after LLC cells were subcutaneously injected into C57BL/6 mice on day 0. Each group was treated either with isotype control (Vehicle) or anti‐PD‐1 mAb or anti‐PD‐1 mAb + anti‐APOC2^K70‐lac^ Ab on days 7, 9, 11, and 13 (four doses), some groups were treated with FX11 daily from day 7, and then on day 17 mice were sacrificed for analysis. B–D) Indicated xenograft tumor cells were collected and photographed (B). Quantification of average tumor weight C) and volume D). Six tumors were included in each group. E) FFAs in tumor interstitial fluid was analyzed in indicated tumors. F–H) TIL prepared from tumor tissue samples on day 17 were subjected to FCM. The frequencies of Foxp3^+^CD4^+^ T cells, the ratio of Foxp3^+^CD4^+^ to CD8^+^ T cells in the TME were examined with FCM (n = 6 per group). Representative contour plots (F), histograms (G), and MFI summaries (H) are shown. I and J) The expression of CTLA‐4, GITR, LAG‐3, and OX‐40 by Foxp3^+^CD4^+^ T cells in the TME was examined with FCM (n = 5 per group). Data represent the mean ± SD of three times of independent experiments. N.S. = not significant, *p < 0.05, **p < 0.01. K) The overall schema of the article.

Because APOC2 in lactylated status is more stable and mainly exists in the tumor interstitial fluid, anti‐APOC2^K70‐lac^ Ab is sufficient to neutralize extracellular APOC2. A previous study showed that FX11 alone inhibits tumor growth.^[^ ^44^ ^]^ However, the therapeutic effect of combination treatment with an anti‐PD‐1 antibody remains unclear. After implantation of LLC cells, anti‐PD‐1 mAb, anti‐APOC2^K70‐lac^ Ab, or FX11 was injected intraperitoneally on the indicated days (**Figure** **8**A). The volume and weight of tumor were significantly decreased in tumors combined with anti‐APOC2^K70‐lac^ Ab or FX11 and anti‐PD‐1 mAb compared with single anti‐PD‐1 mAb treatment (Figure 8B–D). FFAs in the tumor interstitial fluid decreased in tumors treated with anti‐APOC2^K70‐lac^ Ab or FX11 and anti‐PD‐1 mAb (Figure 8E). The frequencies of TNF‐α^+^IFN‐γ^+^ and CD69^+^ tumor‐infiltrating CD8^+^ T cells were increased by anti‐APOC2^K70‐lac^ Ab or FX11 in LLC tumors (Figure S9E–G, Supporting Information), with decreased the frequencies of Treg cells (Figure 8F,G), resulting in a higher ratio of Treg cells: CD8^+^ T cells (Figure 8H) as well as increased expression of their active markers (CTLA‐4, GITR, LAG‐3, and OX‐40) in LLC tumors (Figure 8I,J). In addition, combination treatment with the anti‐APOC2^K70‐lac^ antibody significantly increased the frequency of CD8^+^ T cells among TIL (Figure S9G, Supporting Information). These results suggest that lactylation of APOC2 at K70 plays a major role in mediating the oncogenic effect of FFA in NSCLC, and that anti‐APOC2^K70‐lac^ Ab is an effective enhancer in tumor immunotherapy.

## 3 Discussion

The protein PTMs are key steps in the process of regulating cellular functions and activities, including, but not limited to, immune cell activation and metabolic reprogramming in TME.^[^ ^45^, ^46^, ^47^ ^]^ Protein lactylation on lysine is a recently discovered modification type, and high lactate levels are a specific metabolic byproduct of tumors. Therefore, further research on protein lactylation may significantly improve innovation in cancer therapy. In this study, we present the landscape of protein lysine lactylation and identify 49 histone Kac sites and 2144 non‐histone Kac sites in NSCLC patient samples. Multi‐omics analysis revealed that lactate significantly increased extracellular lipolysis and confirmed the key role of the non‐histone protein lactylated APOC2‐K70 in this pathway, leading to resistance to immunotherapy. Moreover, the mechanisms underlying the regulation of APOC2 lactylation by P300 and HDAC3 were elucidated. It also provides potential therapeutic targets for APOC2‐related diseases.^[^ ^48^ ^]^ Understanding the Kla sites on non‐histone proteins is crucial because they play a significant role in both tumor progression and resistance to immunotherapy.

Under normal physiological conditions, APOC2 is secreted from the liver and intestine and promotes LPL‐mediated lipolysis in capillary beds.^[^ ^33^, ^34^, ^49^ ^]^ However, APOC2 and LPL are markedly upregulated in NSCLC and hepatocellular carcinoma compared to that in normal lung and liver tissues,^[^ ^50^, ^51^, ^52^ ^]^ and LPL significantly reduces the survival time of individuals diagnosed with NSCLC.^[^ ^35^ ^]^ In the high lactic acid microenvironment of NSCLC, lactyl‐APOC2 is paracrinely secreted into TME, binds to highly expressed LPL, and significantly promotes lipolysis in the extracellular space surrounding the tumor cells. Considering the normal expression of p300 and APOC2, as well as lactate metabolism in the liver,^[^ ^53^, ^54^ ^]^ we cannot dismiss the possibility that lactylated APOC2 in liver cells could reach the surrounding tumor tissue alongside LDL/VLDL, which could potentially contribute to immune resistance. However, the significantly elevated levels of APOC2 mRNA expression^[^ ^50^ ^]^ and lactylated APOC2 induced by high lactate level in NSCLC, suggest remarkably increased lipolysis in TME. Thus, this study uncovered a novel mechanism within TME, whereby APOC2 induces lipolysis through paracrine signaling. This process is distinct from the conventional method for APOC2 secretion from liver or intestine.

A range of metabolic by‐products is secreted by various cell types, forming an essential component of TME and promoting tumor proliferation, metastasis, and immunogenicity.^[^ ^55^, ^56^ ^]^ However, the mechanism, type, and function of these secretions remain unknown. Lactate is predominantly generated within tumor cells and secreted from these cells into the TME through MCT4. Once in the TME, lactate serves as an energy source for the proliferation of tumor‐associated immune cells.^[^ ^8^, ^14^ ^]^ Intriguingly, we observed that LDHA was more closely associated with immunotherapy resistance than MCT4 in NSCLC, implying that intracellular lactate also affects immune cells within TME. Lactyl‐APOC2 induced by intracellular lactate secreted from tumor cells and promoted lipolysis in tumor interstitial fluid, thereby leading to Tregs accumulation. Our research uncovered a novel mechanism of lactate regulation in TME, and revealed the complexity of lactate regulation in tumor metabolism. It not only functions as a metabolic product but also exerts non‐metabolic functions via lactylation modifications.

FFAs serve as key intermediates and energy sources in tumor lipid metabolism. Although the interaction between glucose and lipid metabolism has been well‐studied, our research is the first to reveal that lactate, a glycolytic by‐product, directly controls FFA metabolism. We found that lactate stimulates FFA release via the lactylation of APOC2, shedding new light on glucose‐lipid interplay and redefining the role of lactate from waste products to active metabolic regulators. This discovery not only enhances our understanding of tumor lipid metabolism, but also identifies potential targets for immunotherapy. Additionally, an increase in extracellular FFAs leads to higher uptake of FFAs by tumor cells, thereby promoting intracellular FFA accumulation and TG synthesis within tumor cells. TG synthesis in tumor cells may further affect the immunogenicity of tumors or other immune cells through lipid metabolism, thereby enhancing immunotherapy resistance, which presents an intriguing avenue for further research. Given the fundamental role of FFAs in various physiological and pathological processes,^[^ ^57^, ^58^ ^]^ our findings could inform treatment strategies for metabolic disorders such as obesity, diabetes, and cardiovascular diseases.

Therapy resistance and tumor metastasis are the primary causes of cancer‐related deaths in patients with NSCLC. Better diagnostic markers and treatment strategies are required to improve patient outcomes. While the TNM staging system and pathological subtype are used in clinics to predict NSCLC prognosis, they have limitations, particularly in patients with stage II or III NSCLC. Hence, additional prognostic markers are required. In this study, an APOC2 lactyl‐K70 specific antibody was generated, and a positive correlation between lactyl‐APOC2‐K70 and immunotherapy resistance in NSCLC using the antibody was found. Our findings suggest that APOC2 can be used as a biomarker for NSCLC prognosis, although further evaluation in large clinical cohort studies is required. Moreover, we found that Tregs accumulation was the main factor responsible for immunotherapy resistance in NSCLC, and lactyl‐APOC2‐K70 inducing an increase in FFA levels plays a key role in it. Anti‐APOC2^K70‐lac^ Ab significantly reduced the levels of FFAs in TME and enhanced the effectiveness of anti‐PD‐1 antibody treatment. These data demonstrate APOC2‐K70 lactylation serves as a potential biomarker for tumor progression and NSCLC prognosis, and anti‐APOC2^K70‐lac^ Ab has a very promising prospect in NSCLC immunotherapy.

Our study reveals the landscape of protein lysine lactylation and identifies lactyl‐APOC2‐K70 as a key player in augmenting immunotherapy. We demonstrated that lactyl‐APOC2 enhances the stability of itself in tumor cells, triggers the release of FFAs from TG, and promotes tumor metastasis, immune resistance, and T‐cell accumulation. We also found that using an Anti‐APOC2^K70‐lac^ antibody can neutralize extracellular APOC2, and when paired with anti‐PD‐1 treatment, it significantly inhibited tumor growth (Figure 8K). Therefore, combining lactyl‐APOC2‐K70 inhibition with immune checkpoint inhibitors may offer a new and efficacious immunotherapeutic strategy for patients with lung cancer.

## 4 Experimental Section

### Cell Lines, Plasmids, and Reagents

Lewis lung carcinoma (LLC), H1299, Hela, and 293T cells were purchased from the American Type Culture Collection (Rockefeller, MD, USA). All cell lines were maintained in RPMI medium supplemented with 10% fetal bovine serum (FBS; Biosera, Orange, CA). Full‐length Wild‐type (WT) and site‐specific mutants of APOC2 were cloned into the pCDNA 3.0 vector or pLVX‐IRES (lentiviral expression vector) by standard cloning methods. The pLKO.1 control (shN), was generated with control oligonucleotide GCTTCTAACACCGGAGGTCTT. APOC2‐shR was generated with Sh1: CTCCCAACTCTAGCCTGAATT (Located in UTR) or Sh2:CCGCTGTAGATGAGAAACTCA. The mouse APOC2 (wild‐type, K67R, or K67E mutation)‐ expressing LLC cell lines were established via retroviral transduction using a pMMLV‐neo vector (VectorBuilder, Chicago, IL) and growth from a single clone. APOC2‐shR in mouse cells was generated with Sh: GCAGGGCTCCCTCTTAAGTTA. Lenti‐MUS‐APOC2‐WT, ‐K67R, and ‐K67E plasmids contained underlined mutated nucleotides (GCAGGGAAGTTTACTCAGTTA) to generate shRNA‐resistant APOC2 mutants. CD36‐shR was generated with Sh1: CCGACGTTAATCTGAAAGGAA or Sh2: GCCATAATCGACACATATAAA; LDHA‐shR was generated with Sh1: CCACCATGATTAAGGGTCTTT or Sh2 CGTTTGAAGAAGAGTGCAGAT; MCT4‐shR was generated with Sh1: GCTCATACAGGAGTTTGGGAT or Sh2 GCTCATCATGCTGAACCGCTA. LPL‐SiRNA: CCTAACTTTGAGTATGCAGAA or GCCTGAAGTTTCCACAAATAA; APOA1‐SiRNA: CAGCTAAACCTAAAGCTCCTT or GACTATGTGTCCCAGTTTGAA; APOA2‐SiRNA: CTGGTTAACTTCTTGAGCTAT or CTGCAGCCTTGAAGGAGCTTT. Pyruvic acid (HY‐Y0781), Sodium oxamate (HY‐W013032A), and Puromycin (HY‐15695), A‐485 (HY‐107455), KAT8‐IN‐1 (HY‐W015239), NU9056 (HY‐110127), Butyrolactone 3 (MB‐3, HY‐129039), FX11 (HY‐16214), or Gln‐AMS (HY‐112861) were obtained from MedChemExpress (Monmouth Junction, NJ, USA). The lactate concentration was real‐timely monitored using LiCellMo (PHCbi, Janpan).

Used antibodies: Anti‐ APOC2 (1:2000, HPA055997, Sigma‐Aldrich; 1:500, ab172620, Abcam), Flag (1:5000, F1804; Sigma–Aldrich), lactyl lysine (PTM‐1404RM, PTM BIO), and β‐actin (1:5000, A5316; Sigma–Aldrich) antibodies were used. Mouse CD279(PD‐1) (APC‐CY7; clone 29F.1A12, Biolegend #135224, dilution 1:200), anti‐mouse CD223 (LAG‐3) (PE; clone 2C9B7W, Biolegend #125224, dilution 1:200), mouse CD8 (APC; clone 53–6.7, Biolegend#100712, dilution 1:200), mouse CD8 (BV605; clone 53–6.7, Biolegend#100744, dilution 1:200), mouse CD4 (FITC; clone GK1.5, BD#557307, dilution 1:200), mouse CD3 (Percp‐cy5.5; clone 145‐2C11, eBioscience #45‐0031‐82, dilution 1:400), mouse CD3 (BV650; clone 17A2 BD #740530, dilution 1:400), mouse CD36 (PE/Cyanine7; clone HM36 Biolegend #102615, dilution 1:200), mouse CD44 (BV605; clone IM‐7 Biolegend #103047, dilution 1:200), mouse CD62L (APC‐CY7; clone MEL‐14 BD Biosciences #560514, dilution 1:200), mouse CD69 (FITC; clone H1.2F3 BD #553236, dilution 1:200),mouse IFN‐γ (PE; clone XMG1.2 BD#554412, dilution 1:200), mouse TNF‐a (Percp/Cy5.5; clone MP6‐XT22 Biolegend #5506322, dilution 1:200), mouse FoxP3 (BV421;clone MF‐14, Biolegend #126419, dilution 1:200). mouse CD134 (BV711; clone OX‐40 BD #745449, dilution 1:200), mouse CD357 (GITR) (PE/Cyanine7; clone DTA‐1 Biolegend #126317, dilution 1:200).

The site‐specific antibody of lactylated APOC2‐K70 (in vivo grade) was purchased from the Shanghai HuiOu Biotechnology Co., Ltd. The process of production and purification was operated by the company. In brief, the antibody was raised by immunizing rabbits with the synthetic peptide C‐YLPAVDEK(lactyl)LRDLYSK coupled with keyhole limpet hemocyanin (KLH). Anti‐serum was collected after four immunization doses. The valence of the antibody was detected using competitive ELISA^[^ ^59^ ^]^ with the synthetic peptide (C‐YLPAVDEK(lactyl)LRDLYSK) and an unmodified peptide (C‐YLPAVDELRDLYSK).

### 3D Mass Spectrometry Targeting Total Kla

Fresh tumor tissues from lung cancer patients were used for the mass spectrometry in PTM Bio (Hangzhou, China). Briefly, the samples were promptly washed with 1× PBS buffer and pulverized using Cryo‐PrepTM CP02 to minimize blood protein interference. Then, they were frozen in liquid nitrogen, mixed with lysis buffer, sonicated on ice three times using a high‐intensity ultrasonic processor. The concentration of extracted proteins was quantified using the bicinchoninic acid (BCA) assay kit (Beyotime Biotech, China) and samples were diluted to the same protein concentration. Then, they were further digested using trypsin, and Kla‐modified peptides were enriched using Pan antibody‐based PTM (PTM‐1404, PTM Bio) at 4 °C overnight with gentle shaking. Finally, the eluted segments were amalgamated and dehydrated using a vacuum. The resultant peptides were then desalted with C18 ZipTips (Millipore) for LC‐MS‐MS analysis, adhering to the guidelines provided by the manufacturer.

The search results provide signal intensity values of each peptide segment in different samples (Intensity). Based on this information, the following steps were taken to calculate the relative quantification values of the modification sites: The signal intensity values of the modified peptide segments in different samples (**I**) are subjected to centralization transformation, resulting in the relative quantification values of the modified peptide segments in different samples (**R**). The calculation formula was as follows: where i represents the sample, and j represents the peptide segment. Rij = Iij/Mean (Ij).

### Untargeted Metabolomics

LC‐MS analysis for small molecule metabolites were performed by Luming Biotech (Shanghai, China). Briefly, the cell samples were mixed with a 4:1 methanol/water solution, added with trichloromethane, broken down using an ultrasonic homogenizer for 6 min at 500 W. The homogenized mixtures were subjected to ultrasonic extraction in an icy water bath for 20 min, and were centrifuged at a speed of 13 000 rpm at a temperature of 4 °C for a duration of 10 min. The supernatant from each tube was then dried using a freeze‐concentration centrifugal dryer. The dried samples were rehydrated using a 1:4 methanol/water solution. After vertexing for 30 s, the samples were chilled at 4 °C for 2 min and then centrifuged once more at 13 000 rpm at 4 °C for 15 min. The supernatants were subsequently collected using crystal syringes and filtered through 0.22‐µm microfilters. The filtered supernatants were transferred to LC vials and stored at a temperature of −80 °C until the time of LC‐MS analysis. Quality Control (QC) samples were created by combining portions of each individual sample to generate a comprehensive pooled sample.

### Mouse Xenograft Tumor and Lung Metastasis Model

Female C57BL/6 mice (6‐ to 10‐week‐old females; Cyagen) were used for the in vivo studies. Animal experiments were performed according to the guidelines of the National Institutes of Health and approved by the Animal Care Committee of Shanghai General Hospital. Briefly, LLC cells (2×10^6^) were subcutaneously injected into the dorsal part of the mouse (aged 8–10 weeks). After 7 days, mice were randomly divided into four groups, each of 6 mice, and treated as follows: 1. Vehicle + anti‐Isotype IgG; 2. Vehicle + anti‐PD‐1, 3. APOC2^K70^ ^‐lac^ antibody + anti‐PD‐1, 4. FX11+ anti‐PD‐1. From day 7, tumor volume was measured every 2–3 days, and animal survival rate was recorded every day. Tumor volume was calculated as length ×width× width× 0.5. For APOC2^K70^ ^‐lac^ antibody + anti‐PD‐1 combination treatment, treated with 200 µg anti‐PD‐1 (RMP1‐14; Bio X Cell) and/or with 200 µg APOC2^K70^ ^‐lac^ antibody at days 7, 9,11 and 13. For FX11+anti‐PD‐1 combination treatment, LLC tumor‐bearing mice were treated with anti‐PD‐1 intraperitoneally at days 7, 9, 11, and 13 and/or with 1 mg kg^−1^ FX11 (MedChemExpress) or vehicle intraperitoneally at day 7 and every other day until day 17. Mice with tumor sizes larger than 20 mm at the longest axis were euthanized for ethical considerations. To analyze the effector function of tumor‐infiltrating T cells, mice were euthanized on day 19.

In addition, we tested drug resistance to anti‐PD‐1 antibody therapy in shRNA‐resistant APOC2 K67 mutants LLC mouse models. In our study, stable LLC cell lines (shRNA‐resistant APOC2‐WT or ‐K67R clones) (2×10^6^) were resuspended in 100 µL of PBS and inoculated into the right flank of C57Bl/6 mice under anesthesia with 2.5% isoflurane. In order to inhibit the PD‐1/PDL‐1 interaction, anti‐PD‐1 (RMP1‐14, Bio X cell) or isotype control (clone 2A3, Bio X cell) was injected i.p. at 200 µg per mouse in 100 µL of PBS, 7 days after LLC cells injection. Tumor volume was calculated as the length*width^2^ *0.5.

0.5×10^6^ cells (in a volume of 0.1 ml) that stably produced luciferase were injected into the tail vein of nude mice (four weeks old). After 4 weeks, the generation of in vivo bioluminescence was facilitated using d‐luciferin substrate (sourced from Perkin Elmer), and the bioluminescence signal was standardized against the signal detected immediately post cell injection.

### Tumor‐Infiltrating Immune Cell Isolation and FACS Analysis

For TIL analyses, tumors were collected at 9 days after tumor cell injection. Whole tissue was minced and digested in prepared digestion media (1 mg mL^−1^ Collagenase D and 0.1 mg mL^−1^ DNase I in 1x HBSS) at 37 °C for 30 min. Then tissues were filtered through 70 µm pore nylon cell strainers. Separated cells were treated with 1× ACK lysis buffer to lyse red blood cells. Cell pellets were resuspended in PBS with 0.5% FBS for FACS analysis. Cells were stained with the indicated cell surface markers and fixed/permeabilized using a Fixation/Permeabilization kit (eBioscience). Cells were imaged on a BD Biosciences LSRFortessa and analyzed with FlowJo software. Cell counts were determined using flow cytometry (FCM), and counts per weight were subsequently evaluated. For intracellular cytokine assays, cells were stimulated for 5 h with PMA;100 ng mL^−1^)/ionomycin (2 mg mL^−1^) (Sigma–Aldrich). All in vivo experiments were performed at least twice.

### Tissue Specimens and Patient Information

40 samples of neoadjuvant NSCLC patients were obtained from 2020–2021. A 10% residual viable tumor (RVT) indicates major pathologic response (MPR); A total of 164 paraffin‐embedded samples of primary NSCLC were collected from NSCLC patients from 2012 to 2013 at the Shanghai Pulmonary Hospital. The patients’ relapse‐ free survival (RFS) and overall survival (OS) durations were defined as the period from initial surgery to clinically proven metastasis or recurrence and death, respectively. The median patient follow‐up time was 63 months after surgery. All samples were collected with the approval of the Ethics Committee of Shanghai Pulmonary Hospital (Research Ethics Approval Code: K19‐037Y and K21‐189Y), and informed consent was obtained from all patients.

The groups consisted of 30 fresh‐frozen samples of gastric cancer (GC) and 30 Breast cancer (BC) and adjacent normal tissues obtained from patients who underwent surgery at Shanghai General Hospital (Shanghai, China) between October 2016 and May 2021. The samples were promptly moved to liquid nitrogen and preserved at −80 °C pending protein extraction. All samples were collected with the approval of the Ethics Committee of Shanghai General Hospital (Research Ethics Approval Code: 2022SQ123), and informed consent was obtained from all patients.

### Western Blot and Immunoprecipitation (IP)

The proteins of tumor cells or tissues lysates were extracted using the following reagents: RIPA lysis buffer (Biosharp, Hefei, China) containing 1% protease inhibitor cocktail (MedChemexpress, Monmouth Junction, NJ, USA). The protein concentration was measured utilizing the bicinchoninic acid (BCA) assay kit (Beyotime Biotech, China) and all samples were adjusted to match the same protein concentration. Equal amounts of protein were further analyzed with SDS‐PAGE and western blotting. Immunoprecipitation was performed as previously described.^[^ ^60^ ^]^ In summary, the cultured cells were subjected to lysation on ice for a duration of 30 min using an IP lysis buffer that included a 1% protease inhibitor cocktail (MCE, USA). This was followed by a 10 min centrifugation, after which a 100 µL sample of the total cell proteins was combined with 15 µL of anti‐FLAG M2 magnetic beads (Sigma–Aldrich, St. Louis, MO, USA) and incubated overnight at 4 °C. The resulting precipitate was rinsed three times using the lysis buffer and then heated with SDS‐PAGE loading buffer (NCM Biotech). The supernatant that was obtained was used for immunoblotting with the suitable antibodies.

The intensity of total lactyl‐proteins was quantified, scored, and graded (low, 0–6 points; and high, 7–12 points) as described previously.^[^ ^61^ ^]^ To ensure an unbiased result, the data were collected in a double‐blinded manner.

### siRNA Design

The sequences of siRNAs were listed below: P300, 5′‐CAATTCCGAGACATCTTGAGAdTdT; CBP, CCCGATAACTTTGTGATGTTTdTdT; ACSS2, 5′‐GCTTCTGTTCTGGGTCTGAATdTdT; AARS1, CGATGTCCAGAAACGAGTGTTdTdT; AARS2, 5′‐CCATCATACCTTCTTTGAAATdTdT; KAT6A, 5′‐CCGCTGTCACAGTGTAGTATGdTdT; KAT8, 5′‐CGAAATTGATGCCTGGTATTTdTdT; KAT5, 5′‐TCGAATTGTTTGGGCACTGATdTdT; NMT1, 5′‐CGGAAATTGGTTG‐GGTTCATTdTdT; NMT2, 5′‐CGAAGTGCTCAAGGAGTTATAdTdT; APOA2, 5′‐CTCCCAACTCTAGCCTGAATTdTdT.

### Assay Measuring the FFAs Concentration

In order to measure the concentration of FFAs in the culture medium, all cell lines were sustained in RPMl medium enhanced with 10% lipids, excluding FBS. The Free Fatty Acid Quantification Kit from Biovision, Zurich, Switzerland, was utilized to evaluate the total concentration of FFAs. The individual species of FFAs concentrations were identified using LC‐MS.

### Migration and Invasion Assay

The migration and invasion assays were performed in 24‐well Boyden chambers (Corning, NY) with filters (8‐µm pore size) pre‐coated with fibronectin (Roche). APOC2 overexpressing cells, APOC2 knockdown cells, or respective control cells were placed into the upper chamber with 0.5 ml serum‐free DMEM (1 × 10^5^ cells per chamber). DMEM containing 10% fetal bovine serum was added to the lower chamber. After 24 h, cells in the lower chamber were fixed with methanol for 5 min at room temperature followed by crystal violet staining. Experiments were repeated at least 3 times.

### Quantitative RT‐PCR

Total RNA was extracted from cells using a RNeasy Plus Mini Kit (Qiagen), and complementary DNA was synthesized by using an All‐in‐one RT SuperMix Perfect RT‐PCR Kit (Vazyme, China). Real‐time quantitative PCR was performed with SYBR Green Master Mix (Vazyme, China) to assess RNA expression levels on an ABI Quantstudio 6 flex system, and the target RNA levels were normalized to those of Actin.

### The Process of Tumor Interstitial Fluid Isolation

The tumors tissues (0.3 −5 grams) were rinsed in 1 x PBS, and blotted on filter paper provided by VWR Radnor, PA (28298‐020). Then, the tumor tissues were placed onto 20 µm nylon filters from Spectrum Labs, Waltham, MA (148134), secured on top of 50 mL conical tubes, and centrifuged for 10 min at 4 °C at 10^6^ x g. Tumor interstitial fluid was collected from the conical tube, frozen in liquid nitrogen and stored at −80 °C until further analysis.

### PBMC Co‐Culture In Vitro Co‐Culture System

PBMCs were successfully separated from peripheral blood of healthy donors using density gradient centrifugation with Ficoll‐Paque (GE Healthcare, Chicago, IL). PBMCs were stimulated with anti‐CD3 (OKT3) mAb and anti‐CD28 mAb (CD28.2) for 72 h, and then co‐culture with indicated tumor cell lines.^[^ ^41^ ^]^ Four group medium mixtures were collected separately and centrifuged at 400×g, 5 min. The sediments (PBMCs) were washed in PBS twice and prepared for the following flow cytometry experiments.

### Statistics and Reproducibility

Statistical analysis was carried out using two‐tailed Student's t test, and the results are expressed as the mean ± standard deviation (SD) of three independent experiments. *P* values are indicated in the Figure legends. Microscopy images shown are representative of at least 5 fields from 3 independent experiments. Western Blot images are representative of 2 independent experiments. All biological and biochemical experiments were performed with appropriate internal negative and/or positive controls as indicated.

## Conflict of Interest

The authors declare no conflict of interest.

## Author Contributions

J.C., D.Z., Y.W., and M.L. contributed equally to this work. C.J., D.Z., Y.W., M.L., Y.Z., T.F., C.X., L.X., and T.Z. performed experiments. C.H., J.Z., L.S., and G.F. interpreted data and wrote the manuscript. Z.L. and C.H. supervised the study and reviewed the manuscript. All authors approved the final version of the manuscript.

## Supporting information

Supporting Information

Supporting Table 1

Supporting Table 2

## Data Availability

The data that support the findings of this study are available in the supplementary material of this article.
