# Supplementary material for: Lactylated Apolipoprotein C‐II Induces Immunotherapy Resistance by Promoting Extracellular Lipolysis
Source: Adv Sci (Weinh). 2024 Jul 9;11(38):2406333. doi: 10.1002/advs.202406333 (PMC11481198; doi:10.1002/advs.202406333)
Supplement: Supplementary file 1 — Supporting Information [file ADVS-11-2406333-s001.docx]

**Supplementary Information**

**Lactylated Apolipoprotein C-II Induces Immunotherapy Resistance by Promoting Extracellular Lipolysis**

**Supplementary Figures 1-9**

**Figure S1**. Related to Figure 1. The correlations between the proteins in lactate metabolism and tumor progression or treatment.

**Figure S2**. Related to Figure 1. Characterization of the lactylome in Lung cancer.

**Figure S3**. Related to Figure 1. Characterization of the lactylome in Lung cancer.

**Figure S4**. Related to Figure 1. The lactylation of non-histone proteins are associated with tumor metastasis and immune tolerance.

**Figure S5**. Related to Figure 2. A metabolomic study of small molecules in NSCLC cells after lactate treatment.

**Figure S6**. Related to Figure 3. Lactate promotes APOC2 lactylation.

**Figure S7**. Related to Figure 4 and 5. P300 promotes APOC2-K70 lactylation and subsequent APOC2 accumulation.

**Figure S8**. Related to Figure 7. Lactyl-APOC2-K70 promotes Tregs augmentation and immunotherapy resistance.

**Figure S9**. Related to Figure 8. Anti-APOC2^K70-lac^ Ab enhances sensitivity to anti-PD-1 treatment via inhibiting lactyl-APOC2.

**Supplementary Tables 1-2**

**Table S1**. Identification of lactylated proteins and lactylation sites by mass spectrometry.

**Table S2**. Identification of upregulated or downregulated lactylated proteins involved in physiological and pathological signaling pathways in samples from patients treated with neoadjuvant therapy.

Figure S1

**Figure S1. Related to Figure 1. The correlations between the proteins in lactate metabolism and tumor progression or treatment.** (**A**) This dataset includes clinical data and treatment protocols for 40 Non-Small Cell Lung Cancer (NSCLC) patients who underwent combined immunotherapy and chemotherapy. Cases with EGFR mutations are excluded. (**B-G**) The staining of MCT1, MCT4, or LDHA in 40 samples of NSCLC was calculated on a scale of 1-12 points according to the staining of IHC in a double-blinded manner. Scores below 6 are labeled as "low", otherwise they are labeled as "high". **p* < 0.05, ***p* < 0.01 by Student’s t-test. (**B**) Representative immunohistochemical staining of MCT1, MCT4, or LDHA in NSCLC containing pairs of tumor and adjacent normal tissues. (**C-G**) Correlation between MCT1, MCT4, or LDHA and patients with major pathologic response (MPR) (**C-E**), tumor metastasis (**F**), or different stages (**G**).

Figure S2

**Figure S2. Related to Figure 1. Characterization of the lactylome in Lung cancer.** (**A**) The characteristic of six pairs of tumor and adjacent normal tissues which were used to detect total protein lactation modifications via a 3D mass spectrometry. Representative immunohistochemical images displaying hematoxylin-eosin staining. (**B**) Principal Component Analysis (PCA) scatterplot illustrating sample clustering. Each point represents a sample, with blue indicating the adjacent normal tissue group and red indicating the tumor tissue group. The x-axis and y-axis correspond to the first principal component (PC1) and second principal component (PC2), respectively. The distinct separation trend suggests significant differences in Kla between the normal tissue and tumor groups. (**C**) The bar chart shown the numbers of matched spectrums, identified peptides, identified sites, modified peptides, and identified lactyl-proteins. (**D**) Detailed presentation of Kla sites on histones.

Figure S3

**Figure S3. Related to Figure 1. Characterization of the lactylome in Lung cancer.** (**A** and **C**) Principal Component Analysis (PCA) scatterplot illustrating sample clustering. Each point represents a sample The x-axis and y-axis correspond to the first principal component (PC1) and second principal component (PC2), respectively. Adenocarcinoma = AC, squamous cell carcinoma= SCC. (**B** and **D**) Histogram indicating upregulated or downregulated lactylated proteins and sites in tumor tissues of AC v.s. SCC (**B**), or untreatment v.s. undergoing neoadjuvant therapy (**C**). (**E**) Histogram presenting the number of up- or down- regulated lactyl-proteins in different pathways, classified according to Gene Ontology (GO) database.

Figure S4

**Figure S4. Related to Figure 1. The lactylation of non-histone proteins are associated with tumor metastasis and immune tolerance.** (**A, G**) Representative western blot showing lactylated histones (≈20 kDa) and non-histone proteins using anti- pan-lactylation and β-actin antibodies in NSCLC (**A**) or breast cancer (**G**) (T) and adjacent tissues (N). Red arrows indicate the samples with inconsistent expression between lactyl-histones and lactyl-non-histones. (**B-F, H-I**) The total Kla levels of histones or non-histones in 40 paired samples of NSCLC (**B-F**) or 30 paired samples of breast cancers (**H** and **I**) were separately and independently calculated on a scale of 1-12 points according to the staining of western blot using Image J in a double-blinded manner. Scores below 6 are labeled as “low”, otherwise they are labeled as “high”. **p* < 0.05, ***p* < 0.01 by Student’s t-test. (**B**) Scatter diagram showing the scores obtained by subtracting the total histone Kla level score from the total non-histone Kla level score. Points with disparities greater than two standard deviations are marked in pink. (**C**) Scatter plot showing the quantitative analyses of total non-histone Kla levels in paired samples of cancers and normal adjacent tissues. (**D-I**) Correlation between the total non-histone Kla level and patients with different stage cancers (**D**), tumor metastasis (**E** and **H**), and major pathologic response (MPR) (**F**). (**I**) Kaplan–Meier plot of Overall Survival of patients with breast cancers stratified by the total non-histone Kla level.

Figure S5

**Figure S5. Related to Figure 2. A metabolomic study of small molecules in NSCLC cells after lactate treatment.** (**A-C**) The small molecule metabolites from H1299 cells which were treated with PBS or lactate (30mM) for 14 h, and then were analyzed by LCMS. (**A**) Pie chart representing the distribution of different types of metabolites in the sample population. Each slice of the pie corresponds to a unique class, with the size of the slice indicating the relative proportion of that class in the population. (**B**) OPLS-DA (Orthogonal Partial Least Squares Discriminant Analysis) score plot illustrating a clear separation between the Ctrl and lactate treatment groups. The x-axis (t [1]) and y-axis (to [1]) represent the predictive and orthogonal components, respectively. The clear separation between the two groups indicates significant metabolic differences in response to the treatment. (**C**) Histogram depicting the related metabolic pathways between Ctrl and lactate treatment groups. The pathways above the red or blue lines were *p* < 0.01 or *p* < 0.05. (**D**) Q-PCR was employed to detect the knockdown of LDHA and MCT4. Mean ± SD, ***p*<0.01, Student’s t-test.

Figure S6

**Figure S6. Related to Figure 3. Lactate promotes APOC2 lactylation.** (**A**) Table showed the lactyl-proteins related to FFAs synthesis in Lipid transport and metabolism via 3D mass spectrometry targeting total lactyl-proteins. (**B-F**) B-y ion matching diagram of APOC2-K52, K61, K70, and K96 ubiquitination and K61 acetylation sites.

Figure S7

**Figure S7. Related to Figure 4 and 5. P300 promotes APOC2-K70 lactylation and subsequent APOC2 accumulation.** (**A**) H1299 cells were treated with lactate (0 mM, 10mM, or 30mM) for 14 h, and Q-PCR analysis was used to detect APOC2 mRNA level. (**B**) H1299 cells were infected with control shRNA or HDAC3-Sh lentiviruses, or transfected with HDAC3-ovexpression plasmids, and then endogenous APOC2 and β-actin were detected by indicated antibodies. (**C**) *In vitro* lactylation and acetylation of APOC2 by SIRT1 or HDAC3. Flag-APOC2 were purified from 293T cells were incubated with or without His-SIRT1 or His-HDAC3 that were expressed in *E. coli* and purified with Ni-NTA beads. (**D** and **E**) Correlation between HDAC3 and APOC2 protein levels in tissues of NSCLC and GC cohort. Statistical data presented in this figure show mean ± SD, **p*<0.05, ***p*<0.01, Student’s t-test. (**F**) H1299 cells were transfected with Flag-APOC2-K61R, or -K61Q for 24h, and then treated with the CHX (50 μg/ml) for the indicated durations. Cell lysates were analyzed by western blot using FLAG, GFP, or β-actin antibodies. (**G**) Q-PCR was employed to detect the knockdown of LPL. Mean ± SD, ***p*<0.01, Student’s t-test. (**H**) Lactyl-APOC2-K70 level were analyzed via western blot using APOC2^K70-lac^ and β-actin antibodies in tumor tissues (T), or paired adjacent tissues (N). The ratio of APOC2^K70-lac^ / total APOC2 protein (APOC2/ Actin) were quantitated using Image J (Fig. S7H).

Figure S8

**Figure S8. Related to Figure 7. Lactyl-APOC2-K70 promotes Tregs augmentation and immunotherapy resistance.** (**A-D**) TILs prepared from tumor tissue samples on day 17 were subjected to FCM. Representative contour plots (left) and summaries (right) (N = 6) of FCM analysis are shown (**A**, CD4 and CD8; **B**, IFN-γ and TNF-α; **C**, CD8 and CD69; **D**, CD44 and CD62L). Data represent the mean ± SD of three times of independent experiments. N.S. = not significant, **p* < 0.05, ***p* < 0.01.

Figure S9

**Figure S9. Related to Figure 8. Anti-APOC2^K70-lac^ Ab enhances sensitivity to anti-PD-1 treatment via inhibiting lactyl-APOC2.** (**A-C**) 40 samples of NSCLC staining with the markers of Treg cells (CD4^+^ and FOXP3^+^), effector T cells (CD8^+^), or B cells (CD19^+^) were calculated on a scale of 1-12 points according to the staining of IHC in a double-blinded manner. Scores below 6 are labeled as “low”, otherwise they are labeled as “high”. **p* < 0.05, ***p* < 0.01 by Student’s t-test. (**A**) Representative immunohistochemical staining Treg cells or cytotoxic immune cells is shown, illustrating either low or high expression. (**B** and **C**) Correlation between Treg cells (CD4^+^ and FOXP3^+^) or of effector cells (CD8^+^ or CD19^+^) with patients with major pathologic response (MPR). (**D-G**) TILs prepared from tumor tissue samples on day 17 were subjected to FCM. Representative contour plots (left) and summaries (right) (N = 6) of FCM analysis are shown (**D**, IFN-γ and TNF-α; **E**, CD8 and CD69; **F**, CD44 and CD62L; **G**, CD4 and CD8). Data represent the mean ± SD of three times of independent experiments. N.S. = not significant, **p* < 0.05, ***p* < 0.01.
